# Splicing Shift of *RAC1* Accelerates Tumorigenesis and Defines a Potent Therapeutic Target in Lung Cancer

**DOI:** 10.1002/advs.202503322

**Published:** 2025-06-23

**Authors:** Yueren Yan, Ning Wang, Bowen Xing, Min Yang, Jun Shang, Yufang Bao, Lixing Xiao, Ningxia Zhang, Yunpeng Ren, Chunnan Liu, Yuting Chen, Han Han, Yunjian Pan, Lei Lv, Wei‐Xing Zong, Hongbin Ji, Changyou Zhan, Zefeng Wang, Haiquan Chen, Yongbo Wang

**Affiliations:** ^1^ Department of Thoracic Surgery and State Key Laboratory of Genetic Engineering Fudan University Shanghai Cancer Center Shanghai 200032 China; ^2^ Institute of Thoracic Oncology Fudan University Shanghai 200032 China; ^3^ Department of Oncology Shanghai Medical College Fudan University Shanghai 200032 China; ^4^ Department of Cellular and Genetic Medicine School of Basic Medical Sciences & Minhang Hospital Fudan University Shanghai 200032 China; ^5^ University of Chinese Academy of Sciences CAS Key Laboratory of Computational Biology Shanghai Institute of Nutrition and Health Chinese Academy of Sciences Shanghai 200031 China; ^6^ Department of Pharmacology School of Basic Medical Sciences & State Key Laboratory of Molecular Engineering of Polymers Fudan University Shanghai 200032 China; ^7^ School of Life Science Southern University of Science and Technology Shenzhen Guangdong 518055 China; ^8^ Department of Respiratory and Critical Care Medicine Center for Oncology Medicine The Fourth Affiliated Hospital of School of Medicine International School of Medicine International Institutes of Medicine Zhejiang University Yiwu 322000 China; ^9^ Zhejiang Key Laboratory of Precision Diagnosis and Treatment for Lung Cancer Yiwu 322000 China; ^10^ Ministry of Education Key Laboratory of Metabolism and Molecular Medicine Department of Biochemistry and Molecular Biology School of Basic Medical Sciences Fudan University Shanghai 200032 China; ^11^ Department of Chemical Biology Ernest Mario School of Pharmacy Rutgers‐the State University of New Jersey Piscataway NJ 08854 USA; ^12^ Key Laboratory of Multi‐Cell Systems Shanghai Institute of Biochemistry and Cell Biology Center for Excellence in Molecular Cell Science Chinese Academy of Sciences Shanghai 200031 China; ^13^ Shanghai Key Laboratory of Medical Imaging Computing and Computer Assisted Intervention School of Basic Medical Sciences Fudan University Shanghai 200032 China

**Keywords:** EGFR, lung cancer, RAC1, RAC1B, splicing dysregulation

## Abstract

Dysregulated RNA splicing has emerged as a pervasive yet understudied feature of cancer. The small GTPase RAC1 undergoes splicing changes in multiple cancers. However, the in vivo functional disparities between the two major RAC1 isoforms, RAC1B and the canonical RAC1A, and their therapeutic implications in cancer remain largely unexplored. Here, *RAC1B* is found to be significantly upregulated in lung adenocarcinoma (LUAD) patients, particularly in those harboring *EGFR* mutations. Through isoform‐specific overexpression and depletion assays in murine and cellular models of *EGFR*‐mutant LUAD, it is revealed that RAC1B, but not RAC1A, promotes LUAD cell proliferation and tumor growth. Mechanistically, RAC1B stabilizes EGFR by inhibiting its lysosome trafficking and degradation. This function is mediated by the specific binding of RAC1B to the guanine nucleotide exchange factor GDS1, which activates RAC1B. The splicing factor RBM10 which is frequently mutated in LUAD is further identified as a negative regulator of *RAC1B*. Importantly, utilizing LUAD patient‐derived organoid and xenograft models, it is demonstrated that targeting *RAC1B* potently suppresses tumor growth and enhances the efficacy of EGFR inhibitors. Together, the findings delineate functional differences and underlying mechanisms of RAC1 isoforms in LUAD tumorigenesis, highlighting a promising therapeutic route via targeting RAC1B for lung cancer.

## Graphical abstract

Dysregulated RNA splicing is an underappreciated molecular feature of cancer. By integrating murine and cellular models with patient specimens of lung adenocarcinoma, this study demonstrates that RAC1B, rather than RAC1A isoform, promotes tumorigenesis and presents a potent therapeutic target for lung cancer. Mechanistically, RAC1B preferentially binds to GDS1, stabilizes EGFR, and is negatively regulated by the splicing factor RBM10.

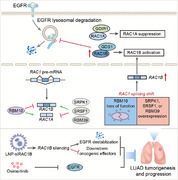

## 1 Introduction

Lung cancer continues to be the leading cause of cancer‐related mortality worldwide.^[^ ^1^ ^]^ Lung adenocarcinoma (LUAD), the most common subtype of lung cancer, exhibits high heterogeneity at cellular and molecular levels. Activating mutations of Kirsten rat sarcoma (*KRAS*) and epidermal growth factor receptor (*EGFR*) are the most prevalent oncogenic drivers in LUAD patients from Western and East Asian populations respectively.^[^ ^2^, ^3^ ^]^ Diverse mutations in tumor suppressor genes and other molecular changes (e.g., epigenetic and RNA processing alterations) co‐occurring with *KRAS* or *EGFR* mutation are considered to play determining roles in augmenting LUAD heterogeneity and complexity.^[^ ^4^, ^5^ ^]^ Intensive mechanistic and translational studies have greatly facilitated the development of new therapeutic approaches that have markedly prolonged the survival of LUAD patients.^[^ ^2^, ^6^ ^]^ Despite these encouraging advancements, the intricate mechanisms underlying LUAD development and progression are still incompletely understood, particularly for *EGFR*‐mutant LUAD.^[^ ^4^, ^7^ ^]^ Moreover, the treatment outcomes for advanced‐stage LUAD patients are still not satisfactory.^[^ ^8^, ^9^ ^]^ Therefore, it is crucial to uncover new molecular mechanisms and develop more efficacious therapeutics for LUAD.

Precursor RNA (pre‐RNA) splicing, a key step of eukaryotic gene expression, is catalyzed by the multi‐megadalton ribonucleoprotein complex known as the spliceosome.^[^ ^10^ ^]^ Alternative splicing (AS) enables the production of functionally distinct splice isoforms from a single gene, a process that occurs in almost all multi‐exon human genes.^[^ ^11^ ^]^ AS is mainly regulated by interactions between numerous regulatory *cis*‐elements within pre‐RNAs and their cognate splicing factors.^[^ ^12^ ^]^ AS regulation plays a critical role in diverse fundamental biological processes.^[^ ^11^ ^]^ Consequently, dysregulation of AS is a major cause of human diseases, and significantly contributes to cancer pathogenesis and therapy.^[^ ^11^, ^13^ ^]^ Altered splicing affects genes involved in nearly every aspect of cancer hallmark, and is being actively pursued as therapeutic targets.^[^ ^13^, ^14^, ^15^ ^]^ However, the functional roles of the majority of splice variants dysregulated in cancer remain poorly understood.

Rac family small GTPase 1 (RAC1) plays vital roles in various cellular processes, such as cytoskeleton reorganization, cell motility, proliferation, and apoptosis.^[^ ^16^ ^]^ RAC1 signaling is tightly controlled at multiple layers including its activity, abundance, and subcellular localization.^[^ ^16^, ^17^ ^]^ Similar to other small GTPase, RAC1 switches between the active GTP‐bound (RAC1‐GTP) and inactive GDP‐bound states in cells. Guanine‐nucleotide exchange factors (GEFs) stimulate the GTP binding and the GDP release, thereby promoting RAC1 activation.^[^ ^17^ ^]^ Conversely, GTPase activating proteins (GAPs) enhance the hydrolysis of GTP to GDP, and GDP nucleotide dissociation inhibitors (GDIs) sequester RAC1 in the cytoplasm and prevent the exchange of GDP to GTP, both leading to RAC1 inactivation.^[^ ^17^ ^]^ Dysregulation of RAC1 signaling has been linked to a variety of human diseases, including cancer.^[^ ^16^ ^]^ Hyperactive RAC1 signaling has been reported to promote tumor development, metastasis, and therapy resistance in different cancer types.^[^ ^18^, ^19^, ^20^ ^]^

**Figure 1:**
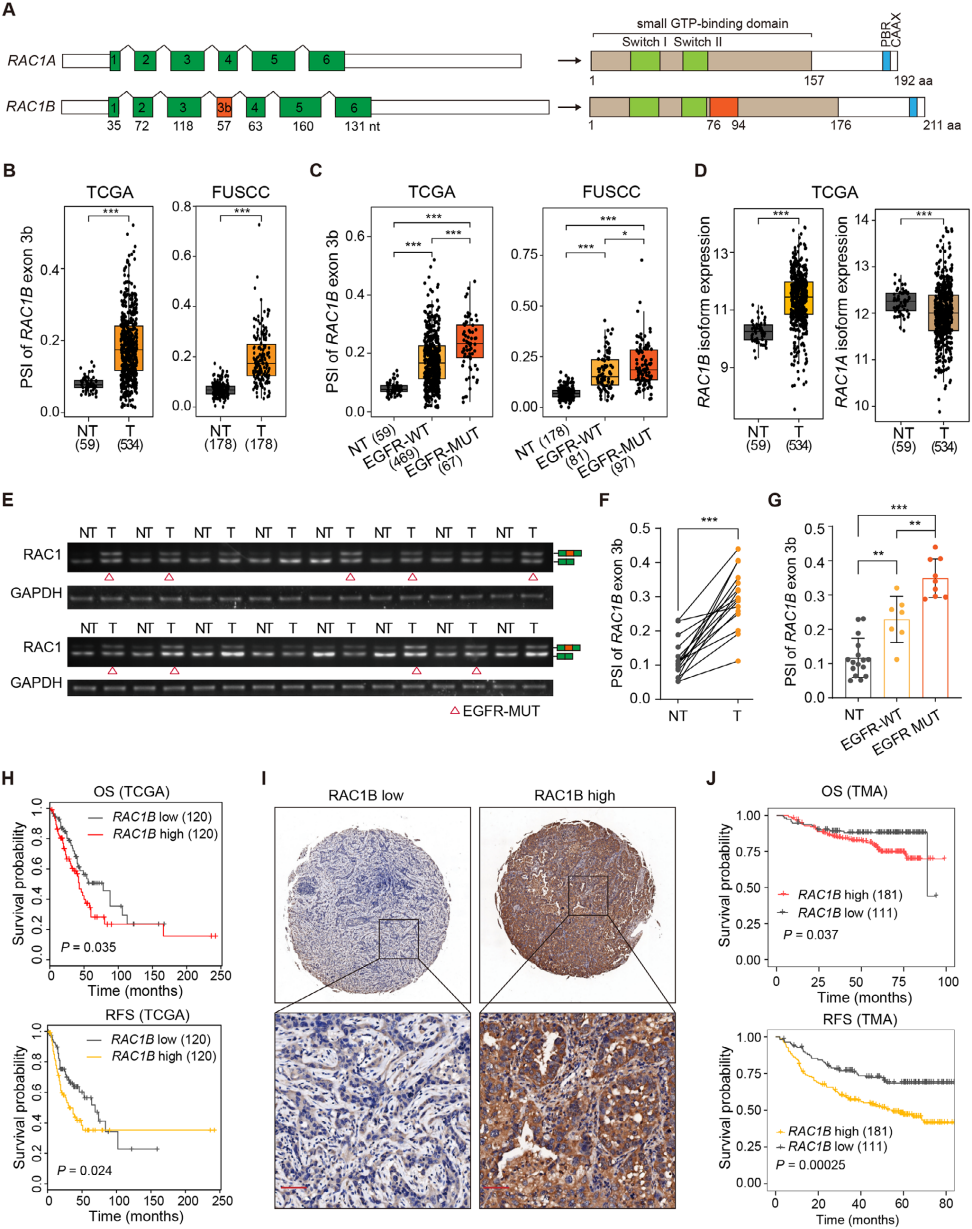
*RAC1B* is elevated and correlates with inferior patient survival in LUAD. A) Schematic illustration of the two *RAC1* splice isoforms produced from alternative splicing of exon 3b. PBR: polybasic region. B) Box plots comparing the percent‐spliced‐in (PSI) levels of *RAC1B* exon 3b between non‐tumor (NT) and tumor (T) tissues of LUAD from the TCGA (The Cancer Genome Atlas, left) and FUSCC (Fudan University Shanghai Cancer Center, right) cohorts. C) Box plots comparing the PSI of *RAC1B* exon 3b among non‐tumor tissues (NT), *EGFR* wild‐type tumors (EGFR‐WT), and *EGFR* mutant tumors (EGFR‐MUT) in LUAD from the TCGA (left) and FUSCC (right) cohorts. D) Box plot comparing *RAC1B* and *RAC1A* isoform expression levels between non‐tumor (NT) and tumor (T) tissues of LUAD from the TCGA cohort. E–G) RT‐PCR validation and quantification of *RAC1B* exon 3b inclusion levels in paired adjacent non‐tumor (NT) and tumor (T) tissues of LUAD with or without *EGFR* mutation. Red triangles(Δ) indicate LUAD patients harboring *EGFR* mutation (EGFR‐MUT). Error bar: ±SEM. H) Kaplan–Meier survival curves for overall survival (OS) and recurrence‐free survival (RFS) in LUAD patients from the TCGA cohort stratified by high and low *RAC1B* expression levels. Log‐rank test. I) Representative immunohistochemical staining images of high and low RAC1B expression estimated by tissue microarray (TMA). Scale bar: 50 µm. J) Kaplan–Meier survival curves for OS and RFS of LUAD patients included in the TMA stratified by high and low RAC1B protein expression. RAC1B levels were estimated by immunoreactive score (IRS, range from 0 to 12). RAC1B Low: IRS 0–6, RAC1B high: IRS 7–12. Log‐rank test. *P < 0.05, ** *P* < 0.01, *** *P* < 0.001, Wilcoxon test in (B–D), Student's *t*‐test in (F), and one‐way ANOVA with Dunnett's multiple comparison test in (G).

Alternative splicing of the fourth exon (designated as exon 3b) in *RAC1* pre‐mRNA generates two splice isoforms. The canonical *RAC1* isoform, termed *RAC1A* hereafter, lacks exon 3b, whereas *RAC1B* contains this exon^[^ ^21^, ^22^ ^]^ (**Figure** **1**A). Exon 3b encodes 19 amino acids that are inserted in‐frame immediately after the Switch‐II domain of RAC1, leading to a conformational change that favors the active GTP‐bound state.^[^ ^23^ ^]^ *RAC1B* has been found to be specifically overexpressed in several cancer types, including lung cancer,^[^ ^21^ ^]^ indicating that RAC1B may have distinct functions compared with RAC1A. Although oncogenic functions of RAC1B have been proposed in breast and colorectal cancer^[^ ^24^, ^25^ ^]^ and implicated in lung cancer,^[^ ^26^, ^27^ ^]^ whether RAC1B and RAC1A distinctly affect tumorigenesis in vivo has not been determined. Furthermore, the functional and therapeutic significance of RAC1B in *EGFR*‐mutant LAUD remains unexplored, and its splicing regulation requires further investigation.

In this study, we establish that RAC1B rather than RAC1A promotes tumorigenesis and elucidate the underlying molecular mechanisms in *EGFR*‐mutant LUAD. Additionally, we uncover the regulatory mechanisms of RAC1B and the therapeutic potential of targeting this oncogenic splicing event. Our findings offer novel mechanistic and clinical insights into the roles of RAC1 and splicing dysregulation in cancer.

## 2 Results

### 2.1 *RAC1B* is Upregulated and Correlates with Inferior Patient Survival in LUAD

To examine the *RAC1* splicing changes in lung cancer, we analyzed the inclusion levels of *RAC1B* exon 3b (percent‐spliced‐in, PSI) using RNA sequencing (RNA‐Seq) data of LUAD patients from TCGA and an independent FUSCC cohort.^[^ ^28^ ^]^ *RAC1B* exon 3b inclusion levels significantly increased in tumor tissues relative to adjacent non‐tumor tissues in both datasets (Figure 1B). Given the previous report regarding *RAC1B* upregulation by the EGFR‐AKT‐SRPK1 signaling,^[^ ^29^ ^]^ we explored the PSI of *RAC1B* exon 3b in *EGFR* mutant (MUT) and wild‐type (WT) LUAD patients. This analysis revealed a more significant increase of *RAC1B* exon inclusion levels in *EGFR*‐MUT than in *EGFR*‐WT samples (Figure 1C). Concordantly, isoform expression analyses using TCGA data showed that, compared to non‐tumor tissues, RAC1B is significantly upregulated while RAC1A is downregulated in tumor tissues (Figure 1D). Additionally, analyses across pathological stages revealed that RAC1B is elevated at all stages with a more profound upregulation in Stages I and II (Figure S1A, Supporting Information), consistent with a previous study.^[^ ^30^ ^]^ In contrast, RAC1A is markedly downregulated in Stages I and II but does not exhibit significant changes in later Stages III and IV (Figure S1A, Supporting Information). The observed increase of *RAC1B* in LUAD was validated by RT‐PCR in LUAD samples (Figure 1E–G).

Survival analyses showed that high expression of *RAC1B* or RAC1A correlates with inferior overall and recurrence‐free survival in patients (Figure 1H; Figure S1B, Supporting Information). These expression patterns and survival correlations suggest the tumor‐promoting function of RAC1B, and a possible role of RAC1A in LUAD progression at advanced stages. To further assess the prognostic value of RAC1B, we performed immunochemistry (IHC) for RAC1B in a tissue microarray (TMA) containing 292 LUAD samples from FUSCC (227out of 292 with *EGFR* mutations, Table S1, Supporting Information). High RAC1B expression in the TMA samples was significantly associated with worse overall survival and recurrence‐free survival (Figure 1I,J). Together, these data indicate the oncogenic function of RAC1B in LUAD, particularly in LUAD patients harboring *EGFR* mutation.

### 2.2 *Rac1b* Rather than Rac1a Accelerates LUAD Development in an *EGFR*‐Mutant Murine Model

**Figure 2:**
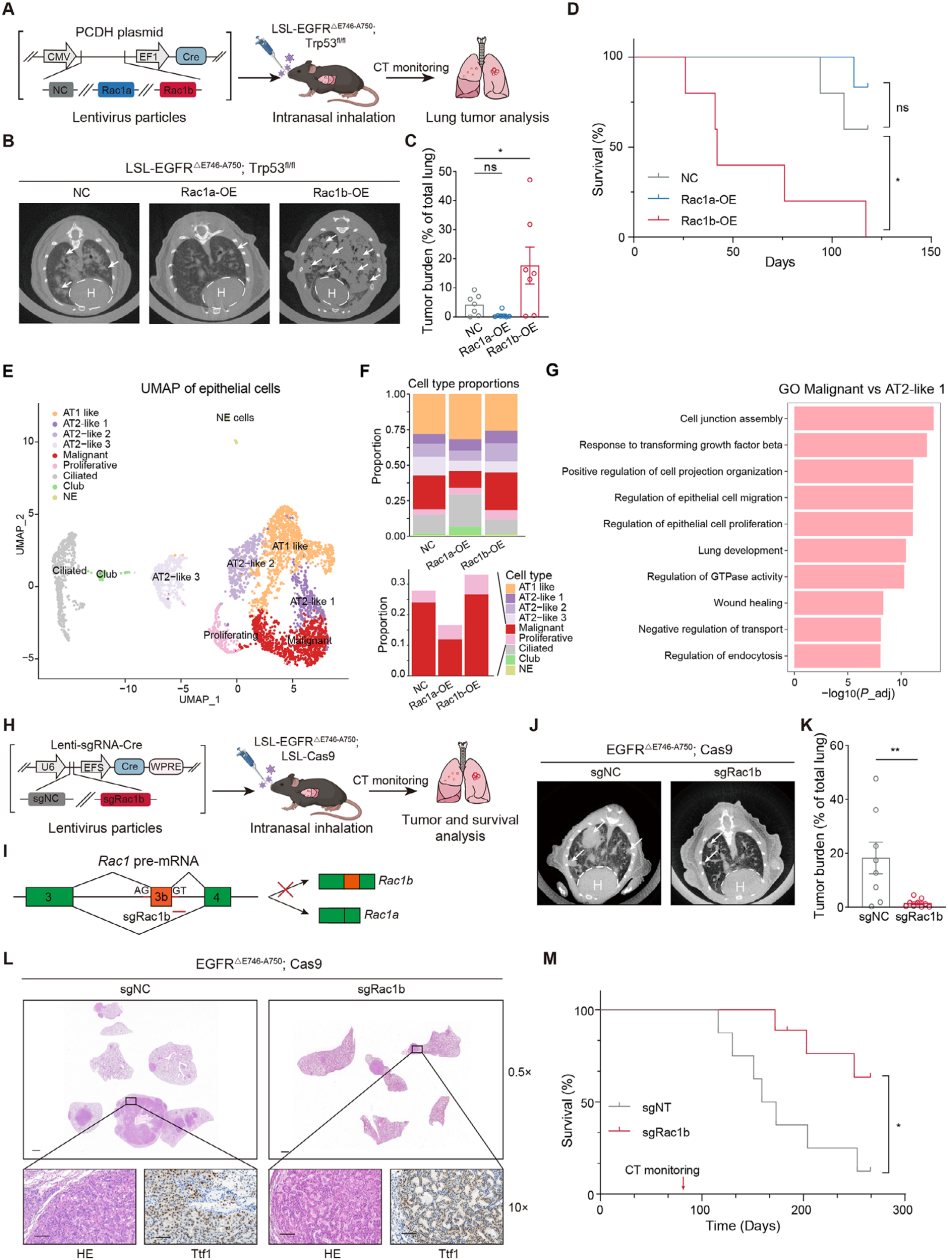
Rac1b but not Rac1a promotes LUAD development in an *EGFR*‐mutant murine model. A) Experimental scheme of lung‐specific overexpression (OE) of Rac1a or Rac1b in LSL‐EGFR^△E746‐A750^; Trp53^fl/fl^ (EP) mice. Lentiviral particles expressing Rac1b or Rac1a and Cre recombinase were intranasally delivered to the lungs of EP mice. Lung tumors were monitored by CT. B,C) Representative images of CT detection (B) and quantification (C) of tumors in the lung of NC (n = 7), Rac1a‐OE (n = 7), and Rac1b‐OE (n = 7) mice. D) Kaplan–Meier survival curves for NC (gray, n = 5), Rac1a‐OE (light blue, n = 6), and Rac1b‐OE (red, n = 5) mice. Log‐rank test. E) UMAP plot of epithelial cells showing distinct cell type clusters. Each color represents a different epithelial cell type. F) Bar plots of cell type proportions across conditions (NC, Rac1a‐OE, and Rac1b‐OE). Upper: Stacked bar plot shows the relative proportion of each cell type in the three conditions, with the total proportion summing to 1 for each condition. Lower: Zoomed‐in bar plot highlighting the specific proportions of selected cell types (Malignant and Proliferative). G) Gene Ontology (GO) enrichment analysis of differentially expressed genes between Malignant and AT2‐like 1 cells. The bar plot shows the selected top 10 significantly enriched GO terms. H) Experimental scheme of lung‐specific depletion of *Rac1b* in heterozygous LSL‐EGFR^△E746‐A750^; LSL‐Cas9 (EC) mice. Lentiviral particles expressing single guide RNA (sgRNA) and Cre recombinase were intranasally delivered to the lung of EC mice. Lung tumors were monitored by micro‐computed tomography (CT). I) Schematic design of sgRNAs specifically targeting *Rac1b*. J,K) Representative images (J) and quantification (K) of lung tumors detected by CT in sgNC (n = 8) and sgRac1b (n = 9) mice. H: heart. L) Representative images of hematoxylin and eosin (HE) and Ttf‐1 immunohistochemistry (IHC) staining of serial lung sections from sgNC or sgRac1b mice. Scale bar = 100 µm. M) Kaplan–Meier survival curves for sgNC (gray, n = 10) and sgRac1b (red, n = 11) mice. Log‐rank test. Error bars represent ±SEM. * *P* < 0.05, ** *P* < 0.01, ns: not significant, one‐way ANOVA followed by Dunnett's tests for comparisons with control in (C), Student's *t*‐test in (K).

To directly interrogate the function of RAC1B relative to RAC1A in vivo, we overexpressed either Rac1a or Rac1b in the lung of LUAD mice models, where the *EGFR* mutant expression and *Trp*53 deletion were induced by Cre recombinase (**Figure** **2**A; Figure S2A, Supporting Information). Computed Tomography (CT) detection and tumor IHC analyses consistently manifested that Rac1b overexpression (OE) significantly accelerated LUAD tumor growth and reduced mice survival compared to control (NC), while Rac1a overexpression showed an opposite trend albeit no statistical significance (Figure 2B–D; Figure S2B,C, Supporting Information). LUAD morphology was confirmed by IHC (Figure S2C, Supporting Information) and the overexpression of Rac1b was assessed by RT‐PCR (Figure S2D, Supporting Information). These results demonstrate the oncogenic function of Rac1b and highlight the disparate roles between Rac1b and Rac1a in LUAD development.

RAC1 has been mostly regarded as a cancer cell‐intrinsic factor involved in the proliferation and migration of cancer cells.^[^ ^18^, ^19^, ^21^ ^]^ To elucidate how Rac1a and Rac1b affect the characteristics of tumor cells, we performed single‐cell RNA sequencing on dissected tumors and analyzed the epithelial cells, which comprise malignant tumor cells and their progenitors. Uniform manifold approximation and projection (UMAP) analysis and subsequent identification according to known marker genes partitioned the cells into nine different clusters (Figure 2E). Intriguingly, the proportion of malignant cells increased in Rac1b‐OE but decreased in Rac1a‐OE compared to NC (Figure 2F), consistent with the changes in tumor growth. In addition, the fraction of the proliferative cells, characterized by high expression of cell division genes and Mki67, also increased in Rac1b‐OE but not Rac1a‐OE tumors (Figure 2F; Figure S2E, Supporting Information). Differentially expressed genes (DEGs) were analyzed between the malignant cells and their proximal AT2‐like 1 cells in the UAMP plot (Figure S2F; Table S2, Supporting Information). Subsequent functional annotation analysis revealed that the DEGs were enriched for cancer‐ and RAC1‐related pathways, including epithelial cell proliferation, cell projection organization, and GTPase activity (Figure 2G; Figure S2G, Supporting Information). These data indicate that Rac1b rather than Rac1a accelerates cell proliferation and tumorigenesis in LUAD.

### 2.3 Depletion of *Rac1b* Impedes LUAD Development in an *EGFR*‐Mutant Murine Model

We next conducted lung‐specific depletion of *Rac1b* in a previously established murine model for *EGFR*‐mutant LUAD,^[^ ^31^ ^]^ utilizing a CRISPR‐Cas9 system with single guide RNA (sgRNA) targeting the splice site of *Rac1b* exon 3b (Figure 2H,I). Strikingly, *Rac1b* depletion (sgRac1b) significantly impeded tumor development compared with control (sgNC), as evidenced by CT scanning (Figure 2J,K; Figure S3A, Supporting Information) and IHC analysis (Figure 2L; Figure S3B,C, Supporting Information), and markedly prolonged mice overall survival (Figure 2M). In sharp contrast to the large tumors dissected in control, only small tumor nodules were observed in the lungs of *Rac1b*‐depleted mice (Figure 2J–L; Figure S3B,C, Supporting Information). IHC analyses of dissected tumors from sgRac1b and sgNC mice confirmed the LUAD morphology, as indicated by the positive staining of LUAD marker protein Ttf1 (Figure 2L). Additionally, the proliferation maker protein PCNA significantly decreased, while no significant change in apoptotic marker protein cleaved caspase 3 was observed (Figure S3D,E, Supporting Information). The substantial reduction of *Rac1b* in sgRac1b tumors was assessed by RT‐PCR (Figure S3F, Supporting Information). Collectively, these results demonstrate that *Rac1b* is critical for *EGFR*‐mutant LUAD development in vivo, reinforcing the observations from *Rac1b* overexpression experiments.

### 2.4 *RAC1B* but not *RAC1A* Promotes Human LUAD Cell Growth In Vitro and In Vivo

**Figure 3:**
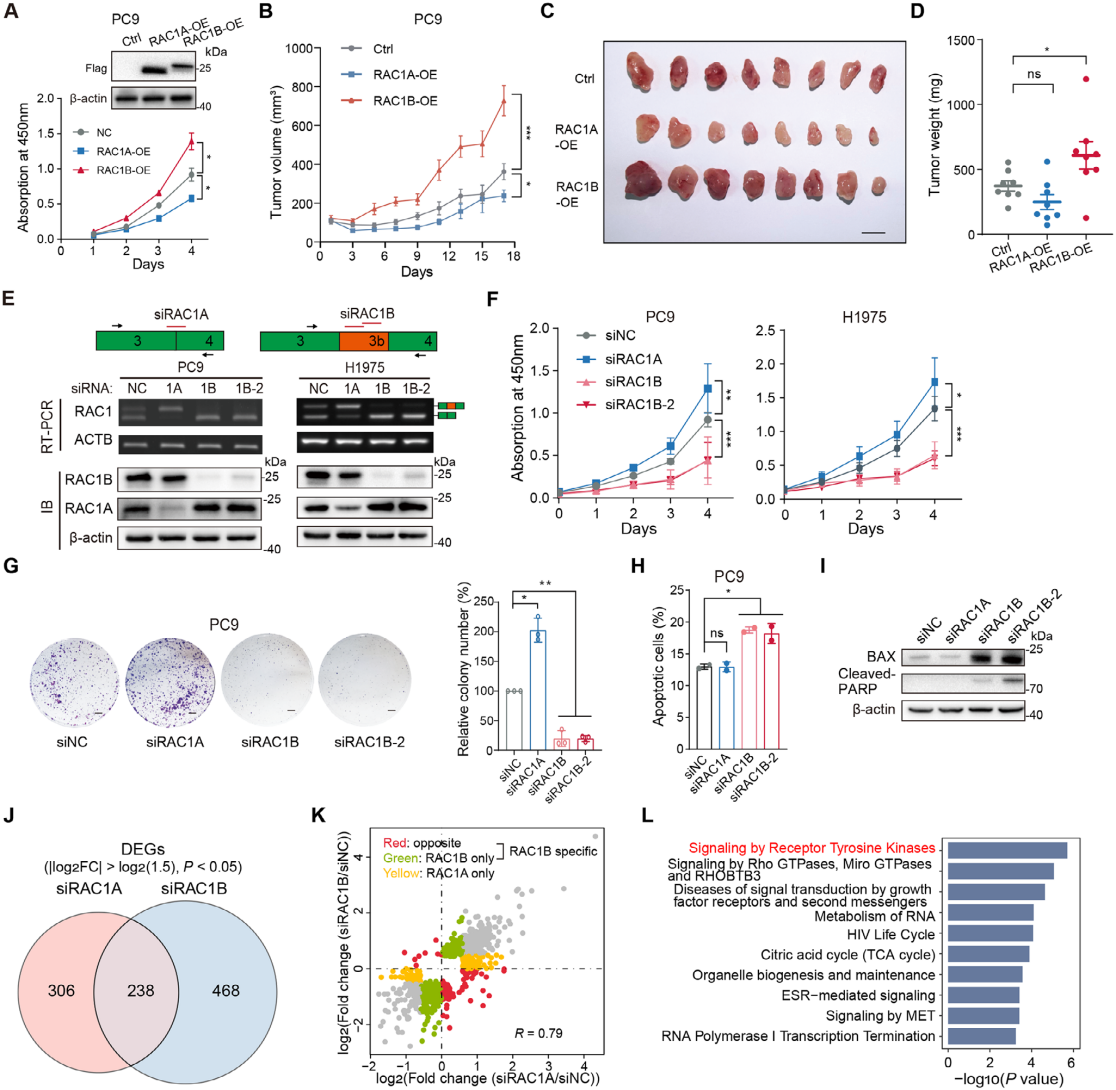
RAC1B rather than RAC1A promotes *EGFR*‐mutant LUAD cell growth and selectively modulates RTK signaling pathway. A) Effects of RAC1A or RAC1B overexpression on cell proliferation of PC9 cells. n = 3 biological replicates. Insert is Western blot analysis of the overexpression (OE) efficiency in PC9 cells stably overexpressing FLAG‐tagged RAC1A (RAC1A‐OE) or RAC1B (RAC1B‐OE). Loading control: β‐actin. B–D) Growth curve (B), the endpoint illustration (C), and tumor weight (D) of xenograft tumors following subcutaneous injection of PC9 cells overexpressing vector control, RAC1A or RAC1B in BALB/c‐nude mice. n = 8 in each group, error bar: ±SEM, scale bar: 1 cm. E) Schematic illustration of small interfering RNAs (siRNAs) targeting *RAC1A* or *RAC1B*, and their knockdown efficiency assessed by RT‐PCR and immunoblotting (IB) in LUAD PC9 and H1975 cells. Red lines indicate siRNAs, and arrows indicate PCR primers detecting *RAC1A* and *RAC1B*. Loading control: ACTB for RT‐PCR and β‐actin for IB. F–I) Effects of *RAC1A* or *RAC1B* silencing on cell proliferation (F), colony formation (G), and apoptosis (H,I) in LUAD PC9 and H1975 cells. n = 3 biological replicates, scale bar: 1 cm, loading control: β‐actin. J) Venn diagram showing the overlap of differentially expressed genes (DEGs) after knockdown of *RAC1A* and *RAC1B* in PC9 cell lines respectively. Cutoff: |log2FC (fold change)| > log2(1.5), *P* < 0.05. K) Scatter plot comparing the fold change of DEGs between *RAC1B* and *RAC1A* knockdown. Genes that are preferentially affected by *RAC1B* knockdown are highlighted in green, those preferentially affected by *RAC1A* knockdown are highlighted in yellow, and those changed in opposite directions in red. L) Bar plot showing the top enriched pathways in genes specifically altered upon knockdown of *RAC1B* compared to *RAC1A* (green and red dots in (K)) using the Reactome database. Error bars represent ±SD unless indicated. * *P* < 0.05, ** *P* < 0.01, *** *P* < 0.001, ns: not significant, two‐way ANOVA with Tukey's multiple comparison tests in (A,B,F), and one‐way ANOVA with Dunnett's multiple comparison tests in (D,G,H).

To further delineate the functions of RAC1A and RAC1B in LUAD, we stably overexpressed RAC1B or RAC1A in the *EGFR‐*mutant human LUAD PC9 cells (**Figure** **3**A). RAC1B overexpression markedly promoted cell proliferation and xenograft tumor growth, whereas RAC1A overexpression exerted opposing effects (Figure 3A‐D), corroborating the findings from murine models. We next specifically silenced each splice isoform in two *EGFR‐*mutant human LUAD cells, PC9 and H1975, respectively (Figure 3E). Knockdown of *RAC1B* inhibited cell proliferation and induced cell death in both cells at day 3 post‐siRNA transfection (Figure 3F–H), with concordantly increased expression of the apoptotic protein BAX and cleaved‐PARP (Figure 3I; Figure S4A, Supporting Information). In contrast, *RAC1A* knockdown led to a moderate increase in cell proliferation but no significant change in cell death (Figure 3F–I). Given that RAC1B is also upregulated in *EGFR*‐wild type LUAD, we examined the effects of *RAC1A* or *RAC1B* knockdown on cell proliferation of *EGFR*‐wild type LUAD A549 and H1944 cells, and obtained changes with similar trends to those in *EGFR*‐mutant cells (Figure S4B,C, Supporting Information). Together, these data manifest that RAC1B, but not RAC1A, promotes LUAD cell proliferation and tumor growth, underscoring distinct functions of the two splice isoforms.

### 2.5 RAC1B Selectively Modulates RTK Signaling Pathway

To elucidate the underlying mechanisms, we performed RNA‐Seq in PC9 cells with *RAC1A* or *RAC1B* knockdown (siRAC1A or siRAC1B). Analysis of the RNA‐Seq data identified genes that that were preferentially upregulated or downregulated in response to *RAC1B* knockdown (Figure 3J,K; Figure S5A,B and Table S3, Supporting Information). Functional annotation revealed that genes preferentially changed in siRAC1B were significantly enriched in receptor tyrosine kinase (RTK) and several other cancer‐related pathways (Figure 3L; Figure S5C, Supporting Information). The expression changes of ‐ several cancer‐related genes were confirmed by reverse transcription and quantitative polymerase chain reaction (RT‐qPCR, Figure S5D,E, Supporting Information), thereby corroborating the reliability of the RNA‐Seq results. These data suggest that RAC1B, in contrast to RAC1A, plays a pivotal role in promoting LUAD cell proliferation and survival at least in part through the modulation of the RTK signaling pathway.

### 2.6 RAC1B Stabilizes EGFR by Attenuating its Lysosome‐Mediated Degradation

**Figure 4:**
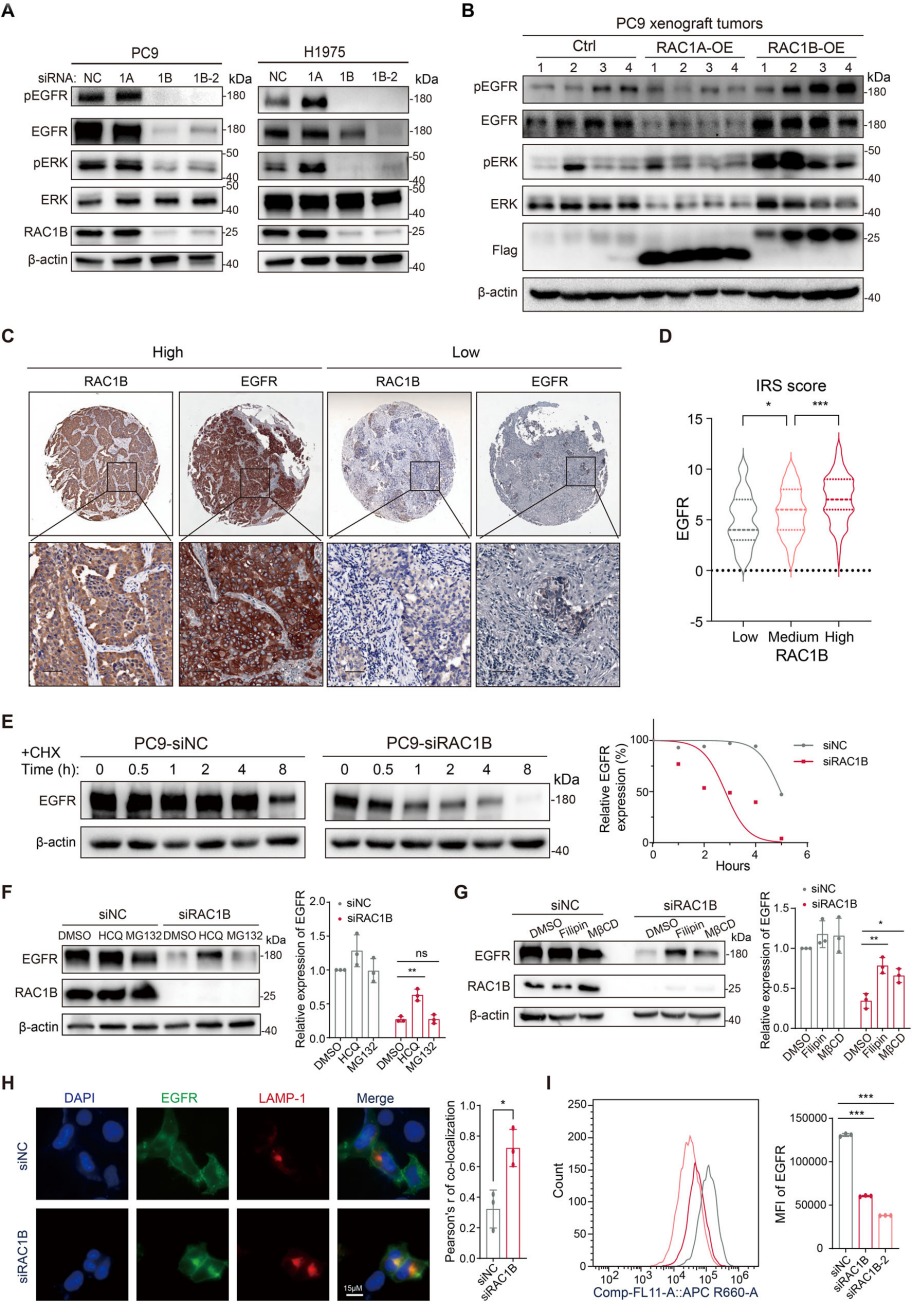
RAC1B stabilizes EGFR by attenuating its lysosome‐mediated degradation. A) Western blot analysis of the phosphorylated and total EGFR and ERK in PC9 and H1975 cell lines following *RAC1A* or *RAC1B* silencing. Loading control: β‐actin. B) Western blot analysis of the phosphorylated and total EGFR and ERK in PC9 xenografts overexpressing FLAG‐RAC1A or FLAG‐RAC1B. Loading control: β‐actin. C) Representative immunohistochemical staining images of RAC1B and EGFR in tumor microarray (TMA). D) Comparison of EGFR expression in RAC1B low (IRS 0–4), medium (IRS 5–8), and high (IRS 9–12) groups. IRS: immunoreactive score. E) Cycloheximide (CHX) chase assay for EGFR degradation in control (siNC) and *RAC1B*‐silenced (siRAC1B) PC9 cells. Cells were treated with CHX (2.5 µg mL^−1^) and the protein expression of EGFR at indicated time intervals was analyzed by Western blot. Quantification of EGFR expression was shown at the right part of the panel. Loading control: β‐actin. F) Western blot analysis of EGFR protein expression in control and *RAC1B*‐silenced PC9 cells treated with DMSO control, HCQ (50 µm, 24 h) or MG132 (10 µm, 24 h). Quantification of EGFR expression was shown at the part of the panel. Loading control: β‐actin. G) Western blot analysis of EGFR protein in control and *RAC1B*‐silenced PC9 cells treated with DMSO control, MβCD (5 mm, 12 h) or Filipin (1 µg mL^−1^, 12 h). Quantification of EGFR expression is shown at the right part of the panel. H) Confocal images of control and *RAC1B*‐silenced HEK293 cells co‐expressing EGFR‐GFP (green) and LAMP1‐mCherry (red). Quantification of colocalized EGFR and LAMP‐1 using the Pearson correlation coefficient was shown at the right part of the panel. n = 3 biological replicates, scale bar: 15 µm. I) Flow cytometry analysis of cell surface EGFR in control and *RAC1B*‐silenced PC9 cells. The mean fluorescence intensity (MFI) of cell surface EGFR was quantified and presented at the right part of the panel. n = 3 biological replicates. Error bars represent ±SD. * *P* < 0.05, ** *P* < 0.01, *** *P* < 0.001, ns: not significant, Student's *t*‐test in (D,H), and one‐way ANOVA with Dunnett's multiple comparison test in (F,G,I).

The oncogenic functions of RTK pathway activation have been well‐established in cancer.^[^ ^32^ ^]^ To identify which RTKs are specifically regulated by RAC1B, we screened changes of 49 phospho‐RTKs following *RAC1B* or *RAC1A* knockdown in the previously established osimertinib‐resistant H1975 cells (H1975‐OR), which exhibited heightened activation of EGFR and downstream EKR signaling,^[^ ^31^ ^]^ using a phospho‐RTK antibody array. Strikingly, phospho‐EGFR (pEGFR) emerged as the sole candidate showing a significant decrease upon *RAC1B* knockdown, whereas it showed negligible change upon *RAC1A* knockdown (Figure S6A, Supporting Information). We then examined phosphorylated and total EGFR with Western blot in *RAC1A* or *RAC1B* knockdown cells, and observed that levels of both considerably declined upon *RAC1B* knockdown (**Figure** **4**A; Figure S6B, Supporting Information). Additionally, a prominent increase in EGFR was detected in RAC1B‐OE but not RAC1A‐OE xenograft tumors (Figure 4B). Phosphorylated‐ERK (pERK), a key downstream effector of EGFR, exhibited concordant changes (Figure 4A,B). Additionally, RAC1B positively correlated with EGFR protein level in human LUAD samples, as determined by IHC (Figure 4C,D). Furthermore, EGFR re‐expression largely reversed the reduced cell proliferation resulting from *RAC1B* knockdown in PC9 cells (Figure S6C, Supporting Information). These data suggest that RAC1B enhances EGFR protein expression and EGFR signaling, thereby promoting LUAD cell proliferation and survival.

Given no significant change in *EGFR* RNA levels upon *RAC1B* knockdown (Figure S5E, Supporting Information) and a previously reported effect of RAC1B on EGFR lysosomal sorting,^[^ ^25^ ^]^ we hypothesized that RAC1B regulates EGFR protein by modulating its trafficking and/or degradation.^[^ ^33^ ^]^ In support of this hypothesis, the EGFR half‐life significantly decreased upon *RAC1B* knockdown in PC9 cells compared to the control (Figure 4E). We then treated the *RAC1B* knockdown PC9 cells with the lysosomal inhibitor hydroxychloroquine (HCQ) or the proteasome inhibitor MG132. HCQ but not MG132 largely increased the EGFR protein level in *RAC1B* knockdown cells (Figure 4F), indicating lysosome‐mediated degradation of EGFR.

Non‐clathrin endocytosis (NCE) is known to preferentially target RTKs to lysosome degradation.^[^ ^34^ ^]^ We thus treated PC9 and A549 cells with inhibitors of NCE, methyl‐β‐cyclodextrin (MβCD) or Filipin, and found that NCE inhibition reverted the decrease of EGFR protein induced by *RAC1B* knockdown (Figure 4G; Figure S6D, Supporting Information). We further examined the co‐localization of exogenously expressed EGFR‐GFP with lysosomal marker LAMP1. The results showed a dramatic increase in lysosome localization of EGFR upon *RAC1B* knockdown in HEK293 cells (Figure 4H). Flow cytometry analysis consistently demonstrated a significant decrease in cell surface EGFR upon *RAC1B* knockdown in PC9 cells (Figure 4I). These lines of evidence indicate that RAC1B prevents lysosomal trafficking and degradation of EGFR, thereby increasing its protein expression.

### 2.7 RAC1B Preferentially Interacts with GDS1 to Stabilize EGFR

**Figure 5:**
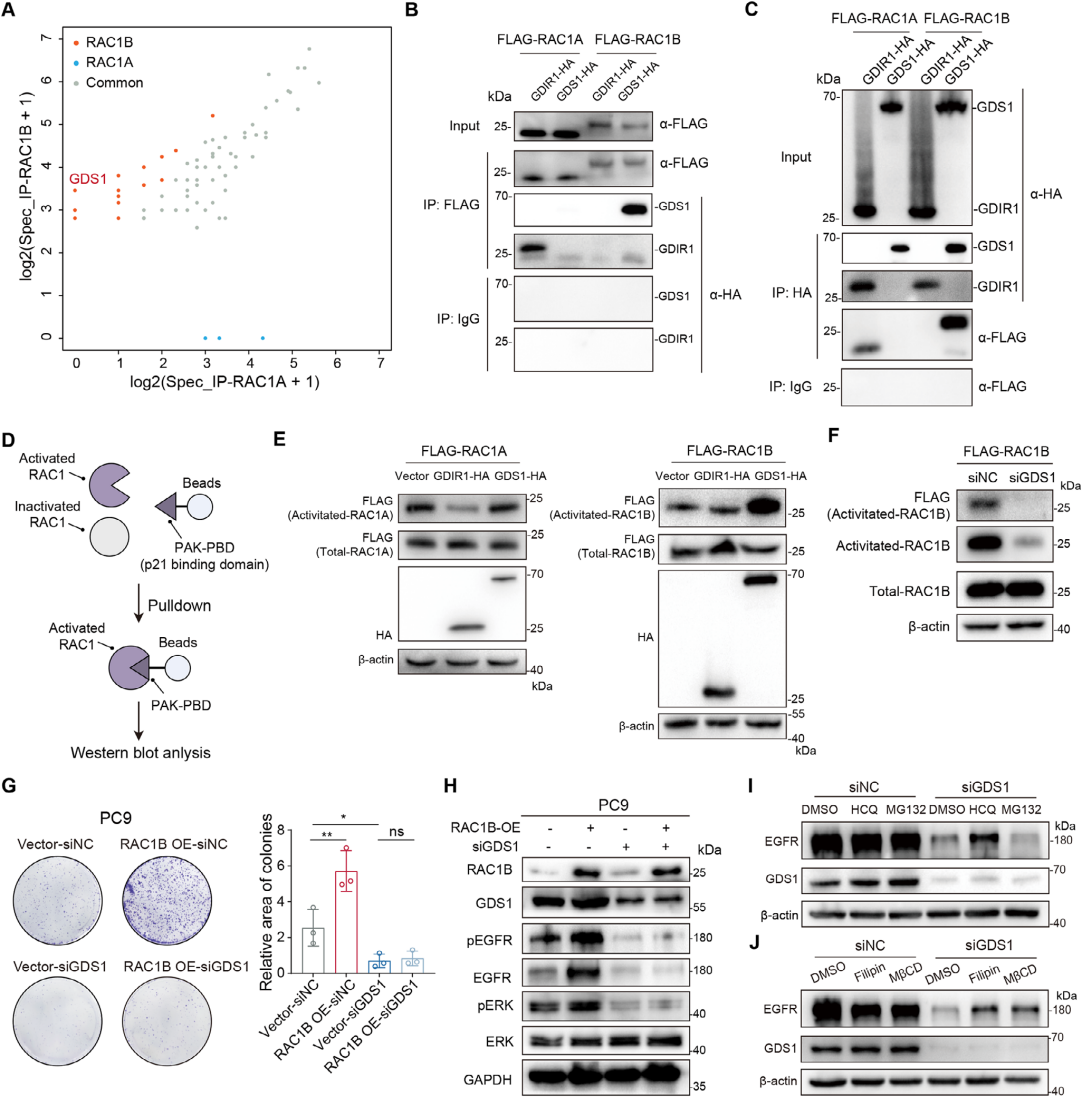
RAC1B preferentially interacts with GDS1 to stabilize EGFR. A) Scatter plot comparing the number of mass spectrums for candidate interacting proteins of RAC1B and RAC1A identified by FLAG antibody immunoprecipitation (IP) and mass spectrometry in PC9 cells stably expressing FLAG‐RAC1B and RAC1A, respectively. B,C) Reciprocal co‐immunoprecipitation and Western blot analyses in PC9 cells stably co‐expressing FLAG‐RAC1A or FLAG‐RAC1B and HA‐GDS1 or HA‐GDIR. Cell lysates were immunoprecipitated with FLAG (B) and HA (C) antibodies respectively, and detected by Western blot with indicated antibodies. IgG antibody serves as the negative control in IP. D) Experimental scheme of RAC1 activation pulldown assay. E) Western blot analyses of PAK‐GST pulldown products in PC9 cells co‐expressing FLAG‐RAC1A or FLAG‐RAC1B and vector control, HA‐GDIR1 or HA‐GDS1. Loading control: β‐actin. F) Western blot analysis of PAK‐GST pulldown products in FLAG‐RAC1B PC9 cells under control or *GDS1* silencing. Loading control: β‐actin. G) Colony formation of PC9 cells upon FLAG‐RAC1B overexpression without or with *GDS1* silencing. Quantification of the colony area was shown at the right part of this panel. Error bar: ±SD, n = 3 biological replicates. * *P* < 0.05, ns: not significant, two‐way ANOVA with Dunnett's multiple comparison test. H) Western blot analysis of indicated proteins in PC9 cells under conditions as described in (G). I,J) Western blot analysis of EGFR protein expression in control and *GDS1*‐silenced PC9 cells treated with DMSO control, HCQ (50 µm, 24 h) or MG132 (10 µm, 24 h) (I) and with DMSO control, MβCD (5 mm, 12 h) or Filipin (1 µg mL^−1^, 12 h) (J), respectively. Loading control: β‐actin.

To delve into mechanisms underscoring the distinct functions of RAC1A and RAC1B, we identified their interacting proteins in PC9 cells over‐expressing FLAG‐tagged RAC1A or RAC1B using immunoprecipitation followed by mass spectrometry (IP‐MS). This assay identified a couple of protein candidates that specifically or preferentially interact with RAC1B and RAC1A (**Figure** **5**A; Table S4, Supporting Information). Notably, the GEF protein GDS1 (encoded by *RAP1GDS1*) emerged as the top candidate specifically interacted with RAC1B (Figure 5A), in agreement with a previous study that showed stronger binding of GDS1 to RAC1B than RAC1A.^[^ ^22^ ^]^ Co‐IP and Western blot assays confirmed the specific interaction between RAC1B and GDS1 (Figure 5B,C), while the known GDI protein GDIR1 (encoded by *ARHGDIA*) of RAC1 preferentially interacted with RAC1A (Figure 5B,C).^[^ ^35^ ^]^ Immunofluorescence assay showed that RAC1A and RAC1B predominantly localize in the cytoplasm and cell membrane in PC9 cells (Figure S6E,F, Supporting Information), which is substantiated by cytosol/nucleus fractionation and immunoblotting (Figure S6G, Supporting Information). Concordant with Co‐IP results, immunofluorescence showed that RAC1B preferentially co‐localizes with GDS1 compared to RAC1A (Figure S6H, Supporting Information).

We next explored whether GDS1 affects RAC1B and RAC1A activation via RAC1 activation assay, in which PAK‐PDB beads bind specifically to the active GTP‐bound, but not the inactive GDP‐bound, RAC1 proteins (Figure 5D). In FLAG‐RAC1A or FLAG‐RAC1B overexpressed PC9 cells that co‐express HA‐tagged GDS1 or GDIR1, the RAC1 activation assays showed that GDS1 specifically activated RAC1B but not RAC1A (Figure 5E). Consistently, *GDS1* silencing remarkably decreased RAC1B activation but had no observable effect on total RAC1B protein (Figure 5F). By contrast, GDIR1 specifically inactivated RAC1A, but had little effect on RAC1B (Figure 5E). These results indicate that isoform‐specific interacting proteins distinctly regulate RAC1B and RAC1A activation.

The observed specific effect of GDS1 on RAC1B activation prompted us to investigate its functional impacts on EGFR stabilization and LUAD cell growth. We silenced *GDS1* in PC9 cells with or without RAC1B overexpression, and found that *GDS1* knockdown alone significantly inhibited cell growth and drastically reduced the protein level of EGFR (Figure S6I,J, Supporting Information). Additionally, the increased cell growth and EGFR protein level upon RAC1B overexpression were abolished by *GDS1* knockdown (Figure 5G,H). Moreover, the decline of EGFR protein upon *GDS1* knockdown was largely reverted by lysosomal inhibitor HCQ and the NCE inhibitors MβCD or Filipin (Figure 5I,J), but not by the proteasomal inhibitor MG132 (Figure 5I). Together, these results indicate that GDS1 stimulates RAC1B activation, thereby preventing EGFR lysosomal degradation and promoting LUAD cell growth.

### 2.8 Systematic Identification of Splicing Regulators for *RAC1B*

**Figure 6:**
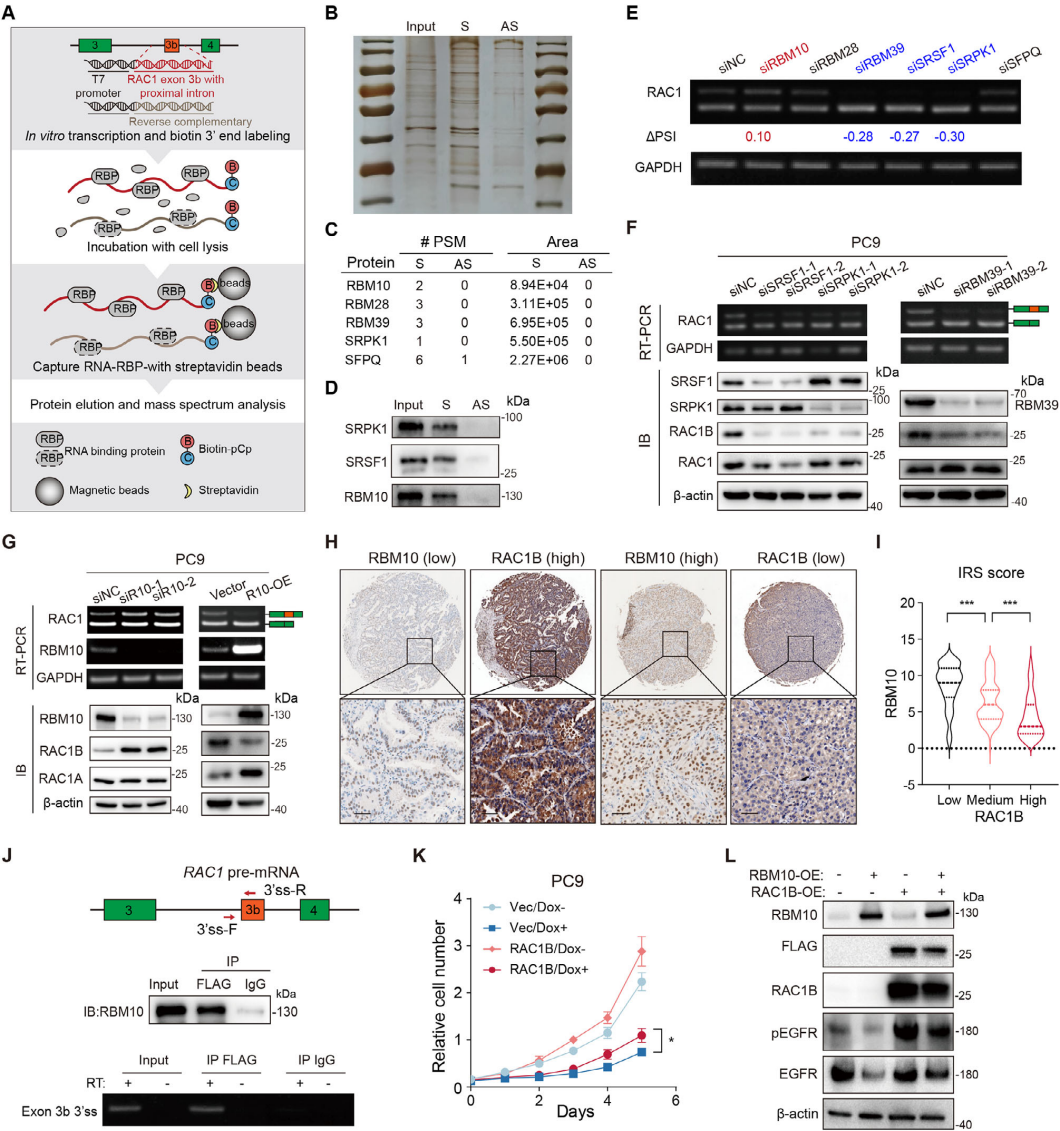
Systematic identification of splicing regulators for *RAC1B*. A) Experimental scheme of RNA pulldown coupled with mass spectrometry detection in PC9 cells. RBP: RNA‐binding protein. B) Silver staining analysis of proteins obtained from RNA pulldown. C) Candidate interacting proteins identified by RNA pulldown assay. S: sense, AS: antisense. D) Western blot confirmation of RBPs that specifically bind to sense RNA. E) RT‐PCR analysis of *RAC1B* exon 3b inclusion in PC9 cells under control or knockdown of the indicated RBPs. |ΔPSI| > 0.1 compared with control was indicated below the gel image. F) RT‐PCR and Western blot analysis of *RAC1B* exon 3b inclusion and protein expression in PC9 cells under control or knockdown of the indicated RBPs. Each RBP was silenced using two individual siRNAs. Loading control: GAPDH for RT‐PCR and β‐actin for Western blot. G) RT‐PCR and Western blot analysis of *RAC1B* exon 3b inclusion and protein expression in PC9 cells with RBM10 knockdown or overexpression. H) Representative immunohistochemical staining images of RAC1B and RBM10. I) Comparison of RBM10 expression among RAC1B low (IRS 0–4), medium (IRS 5–8), and high (IRS 9–12) groups in tumor tissue microarray (TMA). Scale bar = 100 µm. J) CLIP‐PCR confirmation of the binding of RBM10 to the pre‐mRNA region flanking *RAC1B* exon 3b. Primer design was shown at the top, IP efficiency was shown in the middle, and the RT‐PCR detection was shown at the bottom. CLIP: UV crosslinking and immunoprecipitation, ss: splice site. K) CCK‐8 measurement of cell proliferation of PC9 cells overexpressing RBM10 and FLAG‐RAC1B individually or in combination. Error bar: ±SD, n = 3 biological replicates. L) Western blot analysis of indicated proteins under conditions described in (K). * *P* < 0.05, *** *P* < 0.001, Student's *t*‐test in (I), and two‐way ANOVA with Tukey's multiple comparison test in (K).

Although several splicing factors of *RAC1B* have been characterized,^[^ ^36^, ^37^, ^38^ ^]^ the catalog of regulators and regulatory mechanisms in lung cancer awaits further investigation. To systematically identify potential splicing regulators of *RAC1B*, we performed an RNA pulldown assay followed by MS (**Figure** **6**A). RNA sequence spanning *RAC1B* exon 3b and the proximal 150 nucleotide intronic regions was used to pull down the proteins from lysates of nuclear‐fractionated PC9 cells. The corresponding antisense RNA sequence was used as the negative control to minimize background (Figure 6A). Among the several identified putative splicing regulators (Figure 6B,C), SRPK1 has previously been reported to positively regulate RAC1B splicing via SRSF1.^[^ ^36^ ^]^ The interactions between three splicing regulators and the *RAC1B* pre‐mRNA segment were further validated by RNA pulldown and Western blotting (Figure 6D). To ascertain the spicing regulation of *RAC1B* at the endogenous level, we individually knocked down each splicing factor and detected *RAC1B* exon 3b splicing in LUAD PC9 cells. Among these splicing factors, the knockdown of SRPK1, SRSF1, or RBM39 resulted in decreased *RAC1B* exon 3b inclusion and RAC1B protein levels (Figure 6E,F). Further analysis of TCGA LUAD cohort data revealed that the expression levels of SRPK1, SRSF1, and RBM39 significantly upregulated in tumor compared to adjacent non‐tumor tissues, and positively correlated with *RAC1B* levels (Figure S7A,B, Supporting Information). Conversely, the knockdown of *RBM10* increased the *RAC1B* exon 3b inclusion and RAC1B protein levels, while RBM10 overexpression had the opposite effects (Figure 6E,G). Corroborating this negative regulatory axis, the analysis of LUAD TMA revealed a negative correlation between RBM10 and RAC1B protein levels (Figure 6H,I). These results establish novel splicing regulators of *RAC1B* in lung cancer and expand the knowledge of *RAC1B* splicing regulation.

### 2.9 RBM10 is a Critical Suppressor of *RAC1B* Exon 3b Splicing in Lung Cancer

*RBM10* is frequently mutated in LUAD, and its loss‐of‐function (LOF) promotes LUAD development and progression.^[^ ^39^, ^40^, ^41^ ^]^ To further verify the binding of RBM10 to *RAC1B* pre‐mRNA, we performed RNA crosslinking immunoprecipitation and PCR (CLIP‐PCR). This assay showed that RBM10 is specifically bound to the exon 3b‐intron spanning region (Figure 6J). Given the previously reported tumor suppressive role of RBM10 in LUAD,^[^ ^41^, ^42^ ^]^ we proceeded to interrogate whether *RAC1B* contributes to the RBM10 function. We found that the LUAD cell growth inhibition induced by RBM10 overexpression was partially reverted by RAC1B re‐expression (Figure 6K,L), demonstrating the functional significance of RAC1B as a downstream target of RBM10 in LUAD.

### 2.10 RAC1B Inhibition Suppresses Tumor Growth and Improves the Efficacy of Osimertinib in LUAD

**Figure 7:**
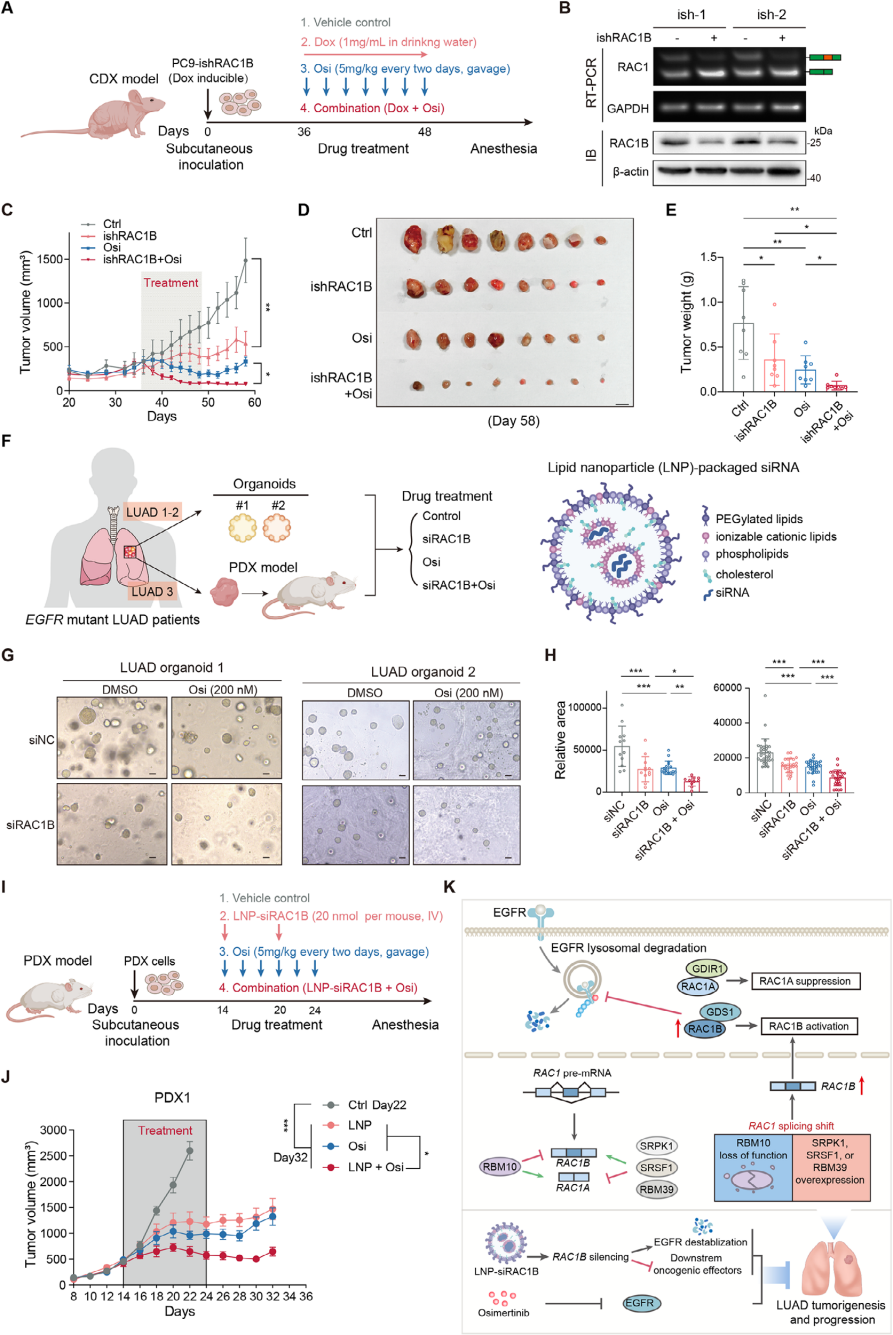
Genetic and pharmacological inhibition of RAC1B potently suppresses LUAD tumor growth and enhances the efficacy of EGFR inhibitors. A) Experimental scheme of xenograft tumor formation and treatment. PC9 cells expressing inducible short hairpin RNA targeting *RAC1B* (ishRAC1B) were subcutaneously transplanted into lower flanks of BALB/c‐nude mice, and the xenograft tumors were subsequently treated with vehicle, doxycycline (Dox, 1 mg mL^−1^ in drinking water), Osimertinib (Osi, 5 mg kg^−1^), or Dox in combination with Osi. B) Knockdown efficiency of ishRAC1b induced by Dox (1 µg mL^−1^, 48 h) in PC9 cells was examined by RT‐PCR and Western blot analysis. Loading control: GAPDH for RT‐PCR and β‐actin for Western blot. C–E) Growth curve (C), endpoint illustration (D) and tumor weight (E) of PC9‐ishRAC1B xenograft tumors. Error bar: ±SEM, scale bar = 1 cm. F) Experimental scheme of constructing patient‐derived organoids (PDOs) and patient‐derived xenograft (PDX) model, drug treatment, and the encapsulation of siRAC1B by lipid nanoparticles (LNP‐siRAC1B). The specimens were obtained from three different lung adenocarcinoma patients with *EGFR* mutation during surgical resections in FUSCC. The schematic formulation of LNP‐siRAC1B is shown at the right part of the panel. G,H) Morphology (G) and quantification of relative areas (H) of PDOs treated with control, siRAC1B and Osi alone or in combination. Scale bar = 50 µm. I) Experimental scheme of PDX construction and treatment. PDX01 was passaged and inoculated in NCG mice, which were subsequently treated with vehicle, or LNP‐siRAC1B (20 nmol per mouse) and Osi (5 mg kg^−1^) alone or in combination. J) Growth curve of PDX01 with treatments as indicated in Figure 7I. The Vehicle control group was terminated at day 22, while the other three groups were terminated on day 34. Error bar: ±SEM. K) Schematic illustration of the oncogenic function and therapeutic value of RAC1B in *EGFR*‐mutant lung cancer. Error bars represent ±SD unless indicated, * *P* < 0.05, ** *P* < 0.01, *** *P* < 0.001, one‐way ANOVA with Dunnett's multiple comparison test in (E,H,J), and two‐way ANOVA with Tukey's multiple comparison test in (C).

Considering the oncogenic and EGFR stabilization function of RAC1B in LUAD, we went on to explore the therapeutic potential of RAC1B in *EGFR*‐mutant LUAD. EGFR tyrosine kinase inhibitors (TKIs) have been widely used to treat advanced‐stage *EGFR*‐mutant LUAD patients, and the third generation EGFR TKI osimertinib (Osi) exhibits superior efficacy than TKIs of previous generations.^[^ ^8^ ^]^ Despite encouraging initial responses, resistance to TKIs, including Osi, inevitably occurs. Therefore, it is critically needed to enhance the TKI efficacy and prevent resistance. Given that EGFR upregulation has been linked to EGFR TKI resistance,^[^ ^9^ ^]^ and a previous study demonstrated that RAC1 inhibition may be effective in EGFR TKI‐resistant LUAD cells,^[^ ^43^ ^]^ we examined the effects of RAC1B inhibition alone or in combination with Osi. We constructed PC9 cells expressing doxycycline (Dox)‐inducible small hairpin RNA (ishRNA) targeting *RAC1B* (**Figure** **7**A,B), which were subcutaneously implanted into the flanks of athymic nude mice. After tumors reached ≈300 mm^3^, the mice were randomized into different groups and treated with vehicle, fed with drinking water containing Dox, treated with osimertinib Osi, or with Dox and Osi in combination (Figure 7A). A single treatment with Dox and Osi resulted in tumor regression, and the combination treatment exhibited significantly stronger inhibitory effects (Figure 7C–E). Notably, after drug withdrawal, tumors rebounded in single treatment groups while did not grow in combination treatment groups (Figure 7C).

To further explore the therapeutic potential of RAC1B, we constructed lipid nanoparticles (LNPs)‐packaged siRNA targeting RAC1B (LNP‐siRAC1B) and examined the efficacy in H1975 cell‐derived xenograft tumors (Figure S8, Supporting Information). Different 2′‐O‐methyl (2′‐OMe) modifications of siRAC1B were synthesized and tested in PC9 cells, and the one that showed strong knockdown efficiency was used for LNP packaging (Figure S8A,B, Supporting Information). To test the effectiveness and toxicity of LNP‐siRAC1B, a low and high concentration was administered to mice via tail vein injection once a week for two weeks when the tumor reached ≈300 mm^3^. We interestingly noticed that the high dose treatment significantly inhibited tumor growth (Figure S8C–E, Supporting Information), and had no significant effects on mice body weight, total protein, and markers of liver and kidney functions in peripheral blood (Figure S8F,G, Supporting Information), while the low dose treatment showed negligible effects on tumor growth (Figure S8C–G, Supporting Information). These data indicate that LNP‐siRAC1B can potently inhibit LUAD tumor growth without severe toxicity.

To strengthen the clinical significance of *RAC1B* inhibition, we constructed two patient‐derived organoids (PDOs) with *EGFR* mutations and performed treatments with siRAC1B and Osi alone or together (Figure 7F; Table S5, Supporting Information). siRAC1B treatment decreased the growth of PDOs, as did Osi treatment, and concurrent treatment exhibited significantly stronger inhibitory effects (Figure 7G,H), agreeing with the results from LUAD cell‐derived xenograft models (Figure 7C–E). We also examined the effects of RAC1 inhibitor EHT1864 because small molecule inhibitor specific for RAC1B is currently lacking,^[^ ^44^ ^]^ and found that EHT1864 treatment also inhibited the growth of LUAD PDO (Figure S8H,I, Supporting Information).

Furthermore, we constructed patient‐derived xenografts (PDXs) in immunodeficient NSG mice, and examined the effects of LNP‐siRAC1B and Osi individually or concurrently in vivo (Figure 7F,I; Table S5, Supporting Information). Remarkably, LNP‐siRAC1B tail‐vein injection treatment retarded tumor growth compared with the control group and showed a concerted inhibitory effect with Osi (Figure 7J; Figure S8J,K, Supporting Information). In addition, the combination treatment effectively prevented tumor regrowth after drug withdrawal compared to a single treatment (Figure 7J). Altogether, these results demonstrate that RAC1B is a promising therapeutic target and that combined RAC1B inhibition strongly improves the efficacy of Osi in LUAD.

## 3 Discussion

Splicing dysregulation significantly contributes to cancer development and progression, but the functional consequences of aberrant splicing events in cancer remain largely uncharacterized. Here we report that RAC1B, but not the conventional *RAC1* isoform RAC1A, promotes tumor development, stabilizes EGFR, and can serve as an ideal therapeutic target in *EGFR*‐mutant LUAD (Figure 7K). Moreover, we identify new splicing regulators of *RAC1B*, deepening the understanding of *RAC1* splicing regulation (Figure 7K).

RAC1 has been intensively studied in different physiological and pathological contexts including cancer.^[^ ^16^, ^19^ ^]^ However, most studies investigated total RAC1 without distinguishing the two splice isoforms, and their functional differences during tumor development have not been evaluated. We demonstrate that RAC1B, rather than RAC1A, is critical for cell proliferation and tumor growth of LUAD both in vitro and in vivo (Figures 2 and 3). Therefore, it is important to delineate the functions of *RAC1* splice isoforms under distinct physiopathological contexts including different tumor types. Aside from the effects on tumor‐intrinsic features, RAC1A or RAC1B may also play a role in other cell types in the tumor microenvironment, such as immune cells, which needs further investigation.

Mutation or elevated expression of EGFR promotes cancer development and is leveraged as a primary target of TKIs.^[^ ^9^, ^45^ ^]^ It is therefore of great interest to elucidate how EGFR is regulated, which is not fully understood despite many investigations.^[^ ^45^, ^46^, ^47^ ^]^ We reveal that RAC1B, via interaction with GDS1, strongly promotes EGFR protein expression by preventing its lysosomal sorting and degradation, highlighting a new regulatory mechanism of EGFR. Considering the significant upregulation of RAC1B (Figure 1) and markedly reduction in cell proliferation as well as EGFR protein levels following *RAC1B* knockdown in both *EGFR*‐mutant and *EGFR*‐wild type LUAD cells (Figures 3F,G and 4A; Figures S4B,C and S6B, Supporting Information), we propose that RAC1B promotes LUAD tumorigenesis at least partly through stabilizing EGFR. In addition to modulating EGFR signaling, we do not preclude the effects of RAC1B on other oncogenic signaling pathways, such as Wnt signaling, in lung cancer (Figure S5C, Supporting Information).

Notably, as *RAC1B* is shown to be positively regulated by the EGFR‐AKT‐SRPK1 axis^[^ ^29^ ^]^ (Figure 1C,G), the RAC1B‐EGFR may form a positive regulatory feedback loop that is postulated to augment *EGFR*‐mutant tumorigenesis and compromise TKI efficacy (Figure 7K). Notably, our study also found that RAC1B inhibition dramatically decreased ERK phosphorylation in LUAD models (Figure 4A,B). This supports that RAC1B inhibition robustly impedes tumor growth and prevents Osi resistance (Figure 7), considering that elevated pERK is known to promote cell proliferation and bypass the effect of EGFT TKI.^[^ ^9^, ^48^ ^]^

We uncovered RBM39 as a new splicing activator and RBM10 as a splicing suppressor of *RAC1B* in LUAD, which greatly facilitates the understanding of *RAC1B* splicing regulation and functional significance in cancer. This complex regulatory network may contribute to the upregulation of *RAC1B* in both *EGFR* mutant and wild‐type LUAD. *RBM10* LOF has been shown to promote lung cancer development and TKI resistance.^[^ ^49^ ^]^ Based on our results, it is conceivable that the increased *RAC1B* splicing resulting from RBM10 LOF is partially responsible for the RBM10‐mediated phenotypes in LUAD, aside from the reported mechanisms.^[^ ^50^ ^]^

Given that *RAC1B* is upregulated in various cancer types while lowly or not expressed in corresponding normal tissues (Figure S9, Supporting Information), it represents a more specific target with a wider therapeutic window than total RAC1. The potential opposing function in tumorigenesis and the broad expression of RAC1A in normal tissues may compromise the effect and increase the toxicity of total RAC1 inhibitors. Our study demonstrated that pharmacological inhibition of RAC1B via systematic administration of LNP‐siRNA effectively suppressed LUAD tumor growth. siRNA‐based therapeutics have been regarded as a very promising realm of new drug development because they can theoretically target any disease‐related genes.^[^ ^51^ ^]^ Groundbreaking achievements have been made in recent years, with several siRNA drugs approved for treating genetic and metabolic diseases, and an increasing number of trials being conducted for cancer treatment.^[^ ^52^ ^]^ Since potent small molecule inhibitors for RAC1 are generally difficult to develop and those specific for RAC1B are currently not available,^[^ ^44^ ^]^ the therapeutic strategy established in our study holds great potential in treating lung cancer and other cancers with RAC1B upregulation.

Aside from RNA‐based therapeutic approaches such as siRNA and antisense oligonucleotide (ASO), future studies are warranted to search for other specific modulators of RAC1B. In this regard, the interaction between GDS1 and RAC1B may be a suitable intervention target for small molecule inhibitors and cell‐permeable peptides, owing to its functional significance in LUAD cell growth. In addition to targeting RAC1B itself, the upstream positive regulators of RAC1B established in our study (e.g., SRPK1 and RBM39) provide alternative therapeutic targets. SRPK1 and RBM39 have been reported to exert oncogenic functions and are actively pursued as therapeutic targets in multiple cancers,^[^ ^53^, ^54^ ^]^ and their inhibitors may also be exploited to downregulate RAC1B.

## 4 Experimental Section

### Mice Studies

EGFR; Cas9 mice (LSL‐hEGFR△E746‐A750 heterozygous and LSL‐Cas9 heterozygous) were described in detail in the previous study.^[^ ^31^ ^]^

EGFR; p53 mice (LSL‐hEGFR△E746‐A750 heterozygous and Trp53 homozygous) were obtained by crossing *EGFR* conditional knock‐in mice with *Trp53* conditional knock‐out mice. Briefly, LoxP sites were inserted flanking the exon 5–7 region of Trp53 via homologous recombination. Trp53 was knocked out by Cre recombinase‐mediated removal of Trp53 exon 5–7 using the Lenti–Cre system. Genotyping primers of EGFR knock‐in mice and Trp53 knock‐out mice were listed in Table S6 (Supporting Information).

For tumorigenicity studies, EGFR; Cas9 mice at 6–8 weeks were randomized into different groups, and were infected intranasally with 5 × 10^6^ TU Lenti‐sgNC‐Cre or Lenti‐sgRac1b‐Cre lentiviruses as described previously.^[^ ^55^ ^]^ 10 weeks after intranasal lentiviral administration, tumors formed in the mice lungs were monitored using a computerized tomography (CT) machine (Perkin Elmer Quantum GXII) and software (Perkin Elmer Analyze12.0) following the manufacturer's manuals. EGFR; Trp53 mice at 6–8 weeks were randomized into different groups, and were infected intranasally with 5 × 10^6^ TU Lenti‐NC‐Cre, Lenti‐mRac1b‐Cre, or Lenti‐mRac1a‐Cre. Eight weeks after intranasal infection, tumorigenicity was monitored by CT. Parameter settings were as follows: voltage 90 kV, current 88 µA, dose 252 mGy, voxel size 72 µm, and high‐resolution scan mode for 4 min.

For survival analysis, the survival end point was determined by evaluating the body condition scores (BCSs)^[^ ^56^ ^]^ in EGFR; Cas9, and EGFR; p53 mice. Mice with a BCS below 2 were euthanized, and the euthanasia date was documented as their final survival date. EGFR; Cas9 mice intranasally inhaled Lenti‐sgCtrl‐Cre or Lenti‐sgRac1b‐Cre and EGFR; p53 mice intranasally inhaled Lenti‐NC‐Cre, Lenti‐mRac1b‐Cre or Lenti‐mRac1a‐Cre were tracked for survival. Survival analysis was performed using the Kaplan–Meier method. Differences among the three groups were evaluated using the log‐rank test. Mice that were euthanized early while still in a healthy state were excluded from the survival percentage calculations.

### Cell Culture

H1975 (RRID: CVCL_1511), and HEK293 (RRID: CVCL_0045) cells were obtained from the Chinese Collection of Authenticated Cell Cultures. PC9 (RRID: CVCL_B260) cell line was purchased from Nanjing Cobioer Biotechnology Co. Ltd. KP cell lines were kindly provided by Dr. Fei Li lab from the School of Basic Medical Sciences, Fudan University. H1975, PC9, and KP cells were cultured in RPMI 1640 (Invitrogen) medium supplemented with 10% FBS. HEK293 was cultured in DMEM (Invitrogen) medium supplemented with 10% FBS. All cultures were maintained under standard culture conditions (37 °C, 5% CO2). Cells were regularly checked for the absence of Mycoplasma infection using PCR with primers targeting Mycoplasma. Cell lines were authenticated by the providers based on short tandem repeat (STR) genotyping.

### Constructs and Lentivirus Packaging

Tet‐on lentiviral plasmid expressing FLAG‐tagged RBM10 (Tet‐FLAG‐RBM10) was constructed as described previously.^[^ ^57^ ^]^ The short hairpin RNAs (shRNAs) specific for RAC1B were cloned into the Tet‐pLKO‐puro vector (RRID: Addgene_21915) linearized by AgeI/EcoRI. Lenti‐sgRac1b‐Cre was constructed by ligating specific DNA oligonucleotides into MluI/NotI linearized Lenti‐sgNT‐Cre plasmid (RRID: Addgene_66895). Lentiviral plasmids expressing FLAG‐tagged human RAC1A (NM_006908), RAC1B(NM_018890), and mouse Rac1a (NM_009007), Rac1b (NM_001347530) were constructed by ligating the corresponding PCR products into NheI/AscI linearized pL‐CMV‐ccdB‐puro vector (Tsingke, Cat. No. PDS271) and XbaI/EcoRI linearized pCDH‐CMV‐EF1‐CRE vector respectively. HA‐tagged GDIR1(NM004309) and GDS1(NM001100427) were cloned into the pLenti‐CMV‐MCS‐HA‐PGK‐BSR vector (OBiO Technology, Cat. No. H13625) linearized by EcoRI/Xbal. GFP‐fused EGFR (NM_005228) was cloned into CMV enhancer‐MCS‐EGFP‐SV40‐Neomycin (Genechem, Cat. No. GV739) linearized by XhoI/AgeI. mCherry‐LAMP1(NM_005561) was kindly provided by Dr. Yang Li lab from the School of Basic Medical Sciences, Fudan University. Oligonucleotide sequences and PCR primers were listed in Table S6 (Supporting Information).

Lentiviral particles were packaged using ViraPower Lentiviral Expression Systems (Thermo Fisher Scientific) following the manufacturer's manual.

### Chemicals

Osimertinib (AZD9291, Cat. No. HY‐15772, CAS No.1421373‐65‐0), CHX (Actidione, Cat. No. HY‐12320, CAS No.66‐81‐9), Hydroxychloroquine (HCQ, Cat. No. HY‐W031727, CAS No.118‐42‐3), MG132 (Cat. No. HY‐13259, CAS No.133407‐82‐6), EHT1864 (Cat. No. HY‐16659, CAS No.754240‐09‐0), and Filipin (Cat. No. HY‐N6716, CAS No.11078‐21‐0) were purchased from MCE Chemicals. Methyl‐β‐cyclodextrin (MβCD, Cat. No. 332615, CAS No.128446‐36‐6) was purchased from Sigma–Aldrich.

### Small Interfering RNA (siRNA)‐Mediated Silencing

The 5 × 10^5^ cells were transfected with siRNA oligos at a final concentration of 20 nm in a 6‐well plate using Lipofectamine RNAi MAX (Thermo Fisher Scientific) according to the manufacturer's instructions. The cells were harvested for RNA and protein analyses as well as phenotypic assays 48 h after transfection unless otherwise specified.

### Cellular Phenotypic Assays

Cellular phenotypic assays were performed as previously described.^[^ ^40^ ^]^ Briefly, cell proliferation rates were examined by estimating viable cell numbers over continuous days using Cell Counting Kit‐8 (Meilunbio) according to the manufacturer's instructions. The oncogenic capacities of cells were estimated by anchorage‐independent colony formation assays. In vitro cell migration was measured by Transwell assays (Falcon, BD). Apoptotic cells were detected using Annexin V‐FITC Apoptosis Detection Kit (Vazyme) and Flow Cytometer CytoFLEX S (Beckman Coulter, FL, USA).

### Reverse Transcription (RT) Followed by Quantitative and Semi‐Quantitative PCR

RNA was extracted using TRIzol reagent (Sigma) according to the manufacturer's instructions. Reverse transcription (RT) was performed using 1 µg of total RNAs and Goldenstar RT6 cDNA Synthesis Kit Ver.2 containing genomic DNA removal module (TsingKe Biological Technology).

Quantitative PCR was performed using AceQ qPCR SYBR Green Master Mix (Vazyme) on ABI Step One Plus system (Applied Biosystems) according to the manufacturer's protocol. The specificity of the PCR amplification was validated by agarose gel electrophoresis and melting curve analyses. Each condition included three technical replicates. qPCR results were quantified using the ΔΔCt method. Sequences of PCR primers are listed in Table S6 (Supporting Information).

Splicing outcomes for specific exons were assessed by RT‐PCR using specific primers binding to the flanking exons, which detect both exon‐included and ‐skipped transcripts. Percent‐spliced‐in (PSI) was defined as: intensity of the exon‐included band/(intensity of the exon‐included band + intensity of the exon‐excluded band) × 100%. Band intensities of RT‐PCR products were calculated as described previously.^[^ ^39^ ^]^

### Western Blot

Western blot analysis was performed as previously described.^[^ ^57^ ^]^ Briefly, cell lysates were prepared with RIPA buffer, and the total proteins were quantified by BCA Protein Assay Kit (Millipore), resolved by SDS‐PAGE, and then transferred to PVDF membranes (Millipore). The membranes were blocked with 5% milk in 1x TBST buffer and sequentially probed with the primary antibodies and secondary antibodies coupled to horseradish peroxidase (HRP). Specific protein bands were detected using an enhanced chemiluminescence (ECL) system (Millipore) and visualized by the ChemiDoc XRS+ image system (BioRad). Primary antibodies used in this study:

RAP1GDS1 (1:500 dilution; Proteintech Cat. No. 10377‐1‐AP, RRID: AB_2284927), RAC1B (1:1000 dilution; Millipore, Cat. No. 09–271, RRID: AB_1163493), RAC1 (1:1000 dilution; Millipore, Cat. No. 05–389, RRID: AB_309712), RBM39 (1:1000 dilution; Proteintech, Cat. No. 21339‐1‐AP, RRID: AB_10733352), SRSF1(1:1000 dilution; Abcam, Cat. No. ab38017, RRID: AB_882519), SRPK1 (1:1000 dilution; Abclonal, Cat. No. A5854, RRID: AB_2766604), RBM10 (1:2000 dilution; Sigma–Aldrich, Cat. No. HPA034972, RRID: AB_10602919), EGFR (1:1000 dilution; Proteintech, Cat. No. 18986‐1‐AP, RRID:AB_10596476), pEGFR (1:1000 dilution; CST Cat. No. 2234, RRID: AB_331701), ERK1/2 (1:2000 dilution; Cell Signaling Technology, Cat. No. 4695, RRID: AB_390779), pERK1/2 (1:2000 dilution; CST Cat. No. 9101, RRID: AB_331646), β‐actin (1:5000 dilution; Proteintech, Cat. No. 66009‐1‐Ig, RRID: AB_2687938), GAPDH(1:5000 dilution; Proteintech, Cat. No. 10494‐1‐AP, RRID:AB_2263076), cleaved PARP (1:1000 dilution;Cell Signaling Technology, Cat. No. 5625, RRID: AB_10699459), BAX (1:1000 dilution; Abmart, Cat. No. T40051, RRID: AB_2910262) HA (1:2000 dilution; Abcam Cat. No. Ab9110, RRID: AB_307019), FLAG (1:5000 dilution; Sigma–Aldrich, Cat. No. F3165, RRID: AB_259529)

The following secondary antibodies were used: Goat anti‐rabbit IgG HRP (1:5000 dilution, Abmart Cat. No. M21002, RRID: AB_2713951), Goat anti‐mouse IgG HRP (1:10000 dilution, Abmart Cat. No. M21001, RRID: AB_2713950).

### Phospho‐RTK Array

The Phospho‐RTK Array Kit (R&D Systems, Cat. No. ARY001B) was used to determine the relative levels of tyrosine phosphorylation of 49 distinct receptor tyrosine kinases (RTKs) in osimertinib‐resistant H1975 cell lines according to the manufacturer's protocol. Specific protein spots were detected using an enhanced chemiluminescence (ECL) system (Millipore) and visualized by the ChemiDoc XRS+ image system (BioRad).

### EGFR Flow Cytometry Analysis

Live cells were stained with Alexa–Fluor 488 anti‐EGFR antibody (Santa Cruz, R‐1, Cat. No. sc‐101 AF488, 1:100), and assessed using a Flow Cytometer CytoFLEX S (Beckman Coulter, FL, USA) with the following parameters: excitation light source 488, 638, 405, and 561 nm. Optical parameters: forward scatter (FS), side scatter (SS), and 13 fluorescence detectors. The acquired data was analyzed using FlowJo V10.

### RAC‐GTP Pull‐Down Assay

RAC1A and RAC1B activities were estimated by the ratio of their active GTP‐bound forms to their total protein levels in PC9 cells using Rac1 Pulldown Activation Assay Kit (Cytoskeleton, Cat. No. BK035) following the manufactures’ manual. Briefly, cells were lysed in cell lysis buffer (50 mm Tris pH 7.5, 10 mm MgCl_2_, 0.3 m NaCl, 2% IGEPAL) containing Protease inhibitor cocktail (Cytoskeleton, Cat. No. PIC02), and cell lysates were then incubated at 4 °C with PAK‐PBD Protein GST Beads (Cytoskeleton, Cat. No. PAK02) for 1 h. PAK beads were then washed with 500 µL ice‐cold Wash Buffer. The washed beads were resuspended by 2x Laemmli sample buffer and boiled for 2 min. The levels of precipitated GTP‐bound RAC1A and RAC1B were measured by Western blot assays using specific antibodies, and then normalized to the levels of their total proteins and β‐actin in total cell lysates.

### Immunofluorescence

For immunofluorescence staining, cells seeded on coverslips were washed with PBS and fixed with 4% buffered paraformaldehyde for 60 min and permeabilized with 0.5% Triton X‐ 100 for 15 min. Subsequently, the cells were blocked with 3% BSA for 60 min at 37 °C, and stained with specific primary antibodies followed by corresponding secondary antibodies. Nuclei were stained with DAPI. Images were captured using a confocal fluorescent microscope (Olympus Microsystems, CA, USA). Quantification analysis was performed with the ImageJ software. Pearson's coefficient was used to analyze colocalization between two target proteins.

### Immunohistochemistry Staining

For immunohistochemistry (IHC) analysis, paraffin‐embedded tissue sections were deparaffinized with xylene and hydrated through graded alcohols into water. Antigen retrieval was performed using a citrate buffer (10 mm sodium citrate buffer, pH 6.0) at sub‐boiling temperature for 15 min. The sections were permeabilized with 0.5% Triton X‐100/PBS for 20 min. Endogenous peroxidase activity was blocked with 3% H_2_O_2_ solution for 10 min, followed by washing three times with PBS. Blocking buffer (3% BSA/PBS) was added to the sections and incubated for 30 min. The slides were then incubated with indicated primary antibodies at 4 °C overnight. After washing three times, sections were incubated for 30 min with corresponding secondary antibodies at room temperature. Signals were detected with freshly made DAB substrate solution (ZSGB‐BIO Company, Beijing, China). Sections were then counterstained with hematoxylin, dehydrated, and mounted with coverslips. Images were captured using an Olympus DP72 microscope (Olympus Microsystems, CA, USA) and analyzed by Image‐Pro Plus 5.1.

### Tissue Microarray Analysis

The cohort for IHC staining of tissue microarray (TMA) consists of 292 LUAD patients from Fudan University Shanghai Cancer Center (FUSCC) diagnosed between 2009 and 2015. All these samples were obtained after surgical resection from patients who had not received neoadjuvant therapy. Immunostaining analysis was independently evaluated by two experienced pathologists at FUSCC using an immunoreactive score (IRS). The staining intensity was graded on a 4‐level scale (no staining = 0, weak staining = 1, moderate staining = 2, and strong staining = 3) and the staining extent was graded on a 5‐level scale (no  =  0, <  10%  =  1, 10–50%  =  2, 51–80%  =  3, and  >  80%  =  4). The scores of the staining intensity and extent were multiplied to yield a final IRS score of 0–12.

### Immunoprecipitation and Mass Spectrometry (IP‐MS)

Interacting proteins of target proteins were identified by IP‐MS. Co‐IP and Western blot analyses were performed to verify protein–protein interaction. Briefly, cell lysates were incubated with indicated antibodies conjugated with Protein A/G Magnetic Beads (MedChemExpress, Cat. No. HY‐K0202) at 4 °C overnight. The immunoprecipitated product was washed three–five times, and boiled in SDS loading buffer for 10 min. A small aliquot of the protein elute was separated on SDS‐PAGE gel and assessed by silver staining using Fast Silver Stain Kit (Beyotime, Cat. No. P0017S). The remaining eluent was resolved in SDS‐PAGE, and the protein bands were extracted from the gel and subjected to LC‐MS/MS detection. The interacting protein candidates were defined by cutoff: log2(Area (IP‐target protein/IP‐IgG)) > 2 and log2(No. of spectrum (IP‐target protein/IP‐IgG)) > 2. The identified interacting proteins that differentially bind to RAC1B or RAC1A were defined by | log2(No. of the spectrum (IP‐RAC1B/IP‐RAC1A)) | > 2. The identified proteins were confirmed by Western blot analysis of the protein elutes after IP.

### RNA Pulldown Assay

DNA sequence containing *RAC1B* exon 3b and proximal intronic regions was PCR amplified from the DNA of PC9 cells. The sense and corresponding antisense RNAs were synthesized by in vitro transcription with HiScribe T7 ARCA mRNA Kit (New England Biolabs, Cat. No. E2060S) following the manufacturer's guidelines. The in vitro transcribed RNA was labeled with biotin at the 3′ end using Pierce RNA 3′ End Desthiobiotinylation Kit (Thermo Fisher Scientific, Cat. No. 20160), and the synthesized RNA was purified by Trizol reagent. The labeled RNA was used to capture and enrich specific RNA‐binding proteins with the Pierce Magnetic RNA‐Protein Pull‐Down Kit (Thermo Scientific, Cat. No. 20164). Briefly, the nuclear fractions of PC9 cells were extracted using the Nuclear and Cytoplasmic Protein Extraction Kit (Beyotime, Cat. No. P0028). The streptavidin beads were pretreated with RNA capture buffer, then 1 µg purified RNA was rotated with streptavidin beads and mixed with 30 mg protein from cell lysates at room temperature for 30 min. Beads were then washed three times with ice‐cold 1x TBST buffer and boiled with SDS loading buffer at 98 °C for 10 min. Samples were run on 10% SDS‐PAGE gels, with an aliquot assessed by the Fast Silver Stain Kit (Beyotime, Cat. No. P0017S), and the remaining was subjected to mass spectrometry detection. The binding proteins were identified based on the abundance and number of spectrum representations in MS. The identified proteins were confirmed by Western blot.

### UV Crosslinking (CL), Immunoprecipitation (IP), and RT‐PCR (CLIP‐PCR)

HEK293 cells (2 × 10^7^ cells per immunoprecipitation) were exposed to 254 nm UV light (300 mJ cm^−2^) using a UV cross‐linker (HL‐2000 HybriLinker, UVP) and promptly lysed with immunoprecipitation buffer (25 mm Tris–HCl pH 7.4, 150 mm NaCl, 1% Triton X‐100, 1 mm EDTA, 5% glycerol, Murine RNase Inhibitor, and Protease Inhibitor Cocktail) on ice for 30 min. The lysates were treated with Turbo DNase I (NEB) and clarified by centrifugation. The resulting supernatants were incubated with either anti‐FLAG antibody or IgG‐bound beads (10 µg antibodies conjugated to 100 µL beads; anti‐FLAG (Sigma–Aldrich, Cat. No. F3165); rabbit IgG (Sigma–Aldrich, Cat. No. I5006); Protein A/G Magnetic Beads, MCE, Cat. No. HY‐K0202) and rotated at 4 °C for 8 h. Subsequently, the beads were collected and sequentially washed two times with high‐salt wash buffer (50 mm Tris–HCl pH 7.4, 1 m NaCl, 1 mm EDTA, 1% NP‐40, 0.1% SDS, and 0.5% sodium deoxycholate) and four times with low‐salt wash buffer (20 mm Tris–HCl pH 7.4, 10 mm MgCl2, and 0.2% Tween‐20). The beads were then resuspended in 180 µL of cold proteinase K buffer and incubated at 50 °C for 45 min to release the precipitated RNA fragments. Immunoprecipitation efficiency was validated through Western blot analysis. RNA extraction was carried out using VeZol Reagent (Vazyme), and subjected to reverse transcription using random primers, and subsequent RT‐PCR assays. Negative controls included IgG for FLAG immunoprecipitation and no reverse transcriptase (RT‐) to control for DNA contamination.

### Generation of Patient‐Derived Organoids (PDOs) and Treatment

Human lung adenocarcinoma organoids derived from two patients (LUAD01 and LUAD04) were cultured in the Lung Adenocarcinoma Organoid Kit (bioGenous, Cat. No. K2138‐LA). Clinicopathological characteristics were listed in Table S5 (Supporting Information). Surgical resected lung tumors were scissored into small pieces and digested by Tumor Tissue Digestion Solution (bioGenous, Catalog: K601003). Then the pellet was resuspended with 20 mL ice‐cold PBS buffer and filtered through a 70 µm cell strainer (Corning, Cat. No. 31050000). Then the filtered pellet was resuspended with 200 µL ice‐cold Lung Adenocarcinoma Organoid Media (bioGenous, Cat. No. K2138‐LA) mixed with 200 µL Corning Matrigel Growth Factor Reduced Basement Membrane Matrix (Corning, Cat. No. 354230). A volume of 80 µL mixture was plated in a pre‐warmed 12‐well plate. The plate was incubated for 0.5 h at 37 °C to allow mixture solidification and 1 mL pre‐warmed Lung Adenocarcinoma Organoid Media was added. PDOs were then constructed and passed to perform treatment studies. RAC1B was depleted in PDOs by transfected with 20 nm RAC1B siRNA or NC siRNA for 96 h using lipofectamine RNAiMAX (Invitrogen, Cat. No. 13778030). After 96 h, the media was replaced with fresh media containing 20nm RAC1B siRNA or NC siRNA with or without 200 nm Osimertinib. Morphological images under different experimental conditions were taken 96 h post‐treatment, and were used to assess the organoid area using ImageJ.

### Lipid Nanoparticle (LNP) Packaging and Treatment

LNP was prepared by the process of spontaneous vesicle formation as described previously.^[^ ^58^ ^]^ In brief, SM‐102, DSPC, HO‐PEG2000‐DMG, and cholesterol were dissolved in ethanol and mixed to yield a molar ratio of 50:10:3:38.5. The weight ratio of LNP to siRNA was 14. siRNA was dissolved in 6.25 mm sodium acetate buffer (pH 5), and then mixed with lipid mixture at ≈3:1 (v/v) using a microfluidic equipment (MPE‐L1s, Suzhou Aitesen Pharmaceutical Equipment Co.,Ltd., China). The total mixing flow rate was 16 mL min^−1^.

### Cell‐Derived Xenograft Tumor Formation and Treatment

Cells were resuspended in ice‐cold 100 µL PBS buffer and implanted subcutaneously into the lower flanks of BALB/c‐nude mice. For RAC1A/RAC1B overexpression, 1 × 10^7^ PC9 cells of control or over‐expressing RAC1A/RAC1B were used, and for *RAC1B* knockdown, 1 × 10^7^ PC9 cells inducibly expressing shRAC1B were used. After tumor formation, tumor volume was estimated every 2–3 days with the following formula: (L * W^2)/2, where L was tumor length and W was tumor width measured by calipers. Xenograft tumors, after reaching a size of 200–300 mm^3^, were randomly assigned into different groups for drug treatment studies. For in vivo induction of RAC1B knockdown, mice were fed with water containing doxycycline (1 mg mL^−1^, Sigma–Aldrich, Cat. No. D3072). Osimertinib or vehicle control (0.9% saline) (5 mg kg^−1^) was administered via gavage consecutively for seven times every 2 days.

The 5 × 10^6^ H1975 cells were resuspended in ice‐cold 100 µL PBS buffer and implanted subcutaneously into the lower flanks of BALB/c‐nude mice. H1975 xenograft tumors, after reaching a size of 200–300 mm^3^, were randomly assigned into three groups for LNP‐siRAC1B treatment studies and treated with PBS, low dose (4 nmol per mouse) or high dose (20 nmol per mouse). LNP‐siRAC1B was administered Intravenously twice via tail vein injection every seven days. The potential toxicity of LNP‐siRAC1B was assessed by monitoring the body weight and markers for liver and kidney functions in the peripheral blood.

### Patient‐Derived Xenograft Tumor Formation and Treatment

Patient‐derived xenograft (PDX) models were constructed and used to assess the clinical benefits of targeting *RAC1B*. PDX01 was generated from *EGFR*‐mutant lung adenocarcinoma. Clinicopathological characteristics were listed in Table S5 (Supporting Information). Briefly, fresh lung cancer tissues were sliced into small fragments (3–5 mm^3^) in the DMEM medium. The tissue fragments were subcutaneously implanted into NSG (NOD‐Prkdcscid Il2rgnull) mice via a puncture needle. When growing to 1000 mm^3^, the tumors were dissected and passaged to new NSG mice. The Phase III PDX01 models were randomly assigned into different groups for drug treatment studies. LNP‐siRAC1B (20 nmol per mouse) was administered twice via tail vein injection every seven days. Osimertinib (5 mg kg^−1^) or vehicle control (0.9% saline) was administered via gavage consecutively for seven times every 2 days.

### scRNA‐seq and Analysis

Tumors dissected from control, Rac1a, or Rac1b overexpressing mice were cut into 1mm^3^ pieces and washed three times with 4 mL of cold PBS solution. Then, the samples were then minced and digested enzymatically for 30 min with agitation using the MACS Tumor Dissociation Kit (Miltenyi Biotec, Cat.No. 130‐095‐929), following the manufacturer's instructions. The dissociated cells were filtered through 40 µm cell strainers (BD, Cat.No.352340) and centrifuged at 500 g for 8 min.The cell pellets were resuspended in red blood cell lysis buffer (Tiangen Biotech, Cat.No. RT122‐1) and incubated at 4 °C for 5 min to remove red blood cells. The cells were washed twice with ice‐cold buffer (0.04% BSA in DPBS) and resuspended. The final concentration was adjusted to 1000 cells µL^−1^ (viability ≥ 80%). DNBelab C Series High‐throughput Single‐Cell RNA Library (MGI Tech Co., Ltd., Cat.No. 940‐000519‐00) was utilized for scRNA‐seq library preparation.

Sequencing data were analyzed using Cell Ranger Count (v2.2.0), which mapped reads to the mm10 genome (v2.1.0) to count unique molecular identifiers (UMIs) and genes per cell.^[^ ^59^ ^]^ Quality control (QC) of cells was based on three criteria: 1) 2000–7000 detected genes, 2) < 7.5% mitochondrial UMIs, and 3) < 80 000 total UMIs. After QC, 39,243 cells were retained for downstream analysis.

Gene expression data were normalized with Seurat (v5.1.0),^[^ ^60^ ^]^ scaling all cells to 10,000 transcripts, while mitochondrial gene expression was regressed out during the scaling step. Highly variable genes were identified for analysis using the FindVariableGenes function. Principal Component Analysis (PCA) was conducted on these genes, retaining the top 15 principal components based on an elbow plot. Batch effects were corrected using Harmony,^[^ ^61^ ^]^ specifying “condition” as the integration variable to align cells across conditions in a shared embedding space. Harmony‐corrected embeddings were used for clustering and visualization. Clustering was performed with a shared nearest neighbor (SNN) approach via Seurat's FindClusters function (resolution = 0.8), identifying 20 clusters. The t‐distributed stochastic neighbor embedding (t‐SNE) and uniform manifold approximation and projection (UMAP) embeddings were generated for visualization.

Cell types were annotated based on known marker genes, identifying epithelial, immune, and stromal cell types.^[^ ^62^, ^63^, ^64^ ^]^ From the annotated clusters, 4114 epithelial cells were isolated and further analyzed. This subset was processed with additional normalization, scaling, and dimensionality reduction as described above. Using canonical marker genes, epithelial cell clusters were further subdivided into specific subtypes, including “AT1‐like,” “AT2‐like,” “Malignant,” “Proliferative,” “Ciliated,” “Club,” and “NE” cells.^[^ ^62^, ^63^, ^64^ ^]^ This annotation highlighted tumor‐related cell states, including the “Malignant” and “Proliferative” clusters. The “AT2‐like 1”, which was proximal to the “Malignant” subtype based on UAMP clustering, was used as a baseline for downstream comparisons. Differentially expressed genes (DEGs) were identified within each cluster and between clusters using the FindAllMarkers function, employing a Wilcoxon rank‐sum test.

### RNA‐Seq and Analysis

RNA sequencing was performed as previously described.^[^ ^31^ ^]^ The RNA‐seq reads were mapped to the human genome reference (ENSEMBL genome browser hg38) using STAR version 2.4.2a with default parameters. Gene‐level quantification was performed by RSEM version1.3.3 with the parameter configuration “–paired‐end –alignments,” based on bam files obtained from STAR alignment. This step provided read counts for each gene, which were used in downstream analyses.

Differentially expressed gene analysis was conducted using the “DESeq2” package in R. The analysis compared gene expression levels between experimental groups (e.g., siRAC1A vs siNC, siRAC1B vs siNC). Genes were considered differentially expressed if they met the criteria of |log2FoldChange| > log2(1.5) and a *p*‐value < 0.05, ensuring the selection of genes with significant and biologically relevant expression changes.

Functional enrichment analysis was performed using the Metascape online tool (https://metascape.org/gp/index.html#/main/step1), which integrates multiple pathway databases to identify significantly enriched biological processes and pathways among the differentially expressed genes. The results were visualized through bar plots in R to highlight key pathways and processes influenced by *RAC1A* and *RAC1B* knockdown.

### TCGA and FUSCC Data Analysis

The gene and transcript expression data from TCGA are sourced from Xena Browser (https://xenabrowser.net/datapages/) and GEPIA 2 (http://gepia2.cancer‐pku.cn/#index), while splicing data were obtained from the IDeAS database using Paean.^[^ ^65^ ^]^ The FUSCC dataset, comprising 189 paired LUAD and adjacent non‐tumor tissue samples, was processed using the same pipeline for gene and transcript expression. Splicing quantification was performed using rMATS.^[^ ^66^ ^]^

Survival analysis was conducted using Kaplan–Meier estimates to evaluate the association between *RAC1B* expression levels or PSI levels and patient outcomes, specifically overall survival (OS) and recurrence‐free survival (RFS). The survival curves were visualized using the “survminer” package in R, and *p*‐values < 0.05 were considered indicative of statistically significant differences in survival outcomes.

### Primer and Oligonucleotide Sequences

All primer and oligonucleotide sequences were listed in Table S6 (Supporting Information).

### Study Approval—Patient Tissue Samples

Human lung adenocarcinoma (LUAD) specimens were obtained with the approval of the institutional review board of Fudan University Shanghai Cancer Center, Shanghai, China. All patients underwent surgery and provided informed consent. All cases were re‐reviewed by pathologists for confirmation of tumor histology and tumor content.

### Study Approval—Mice Studies

All the animal experiments were performed in compliance with the NIH Guide for the Care and Use of Laboratory Animals (National Academies Press, 2011) and were approved by the Animal Ethics Committee of the School of Basic Medical Sciences at Fudan University.

## Conflict of Interest

The authors declare no conflict of interest.

## Author Contributions

Y.Y. and N.W. contributed equally to this work and co‐first authors. Y.B.W., H.Q.C., Z.F.W., C.Y.Z., and H.B.J. conceived, designed, and supervised the study. Y.R.Y. performed most of experiments. N.W. and J.S. analyzed the genomics data. B.W.X, M.Y., Y.F.B., N.X.Z., Y.P.R., C.N.L., and Y.T.C. performed part of the experiments. Y.J.P. and H.Q.C. supported the experiments related to clinical samples. L.V., W.X.Z., and H.B.J. contributed methodology. Y.R.Y., N.W., Y.F.B., and Y.B.W. prepared the figures. Y.B.W., Y.R.Y., and N.W. wrote the manuscript. Y.B.W., H.B.J, Z.F.W., W.X.Z., H.Q.C., and C.Y.Z. edited the manuscript. All authors contributed to data interpretation.

## Supporting information



Supporting Information

Supplemental Table 1

Supplemental Table 3

Supplemental Table 4

Supplemental Table 5

Supplemental Table 6

Supplemental Table 7

## Data Availability

The data that support the findings of this study are available in the supplementary material of this article.
