# Supplementary material for: Splicing Shift of RAC1 Accelerates Tumorigenesis and Defines a Potent Therapeutic Target in Lung Cancer
Source: Adv Sci (Weinh). 2025 Jun 23;12(33):e03322. doi: 10.1002/advs.202503322 (PMC12412597; doi:10.1002/advs.202503322)
Supplement: Supplementary file 1 — Supporting Information [file ADVS-12-e03322-s006.pdf]

ADVANCED SCIENCE
Open Access

Supporting Information

for *Adv. Sci.*, DOI 10.1002/advs.202503322

Splicing Shift of *RAC1* Accelerates Tumorigenesis and Defines a Potent Therapeutic Target in Lung Cancer

Yueren Yan, Ning Wang, Bowen Xing, Min Yang, Jun Shang, Yufang Bao, Lixing Xiao, Ningxia Zhang, Yunpeng Ren, Chunnan Liu, Yuting Chen, Han Han, Yunjian Pan, Lei Lv, Wei-Xing Zong, Hongbin Ji, Changyou Zhan, Zefeng Wang*, Haiquan Chen* and Yongbo Wang**

Supporting Information

This PDF file includes:

Supporting Figures. S1 to S9

Other supplementary materials for this manuscript include:

Supplementary Table S1 to S6 (provided as separate excel files)

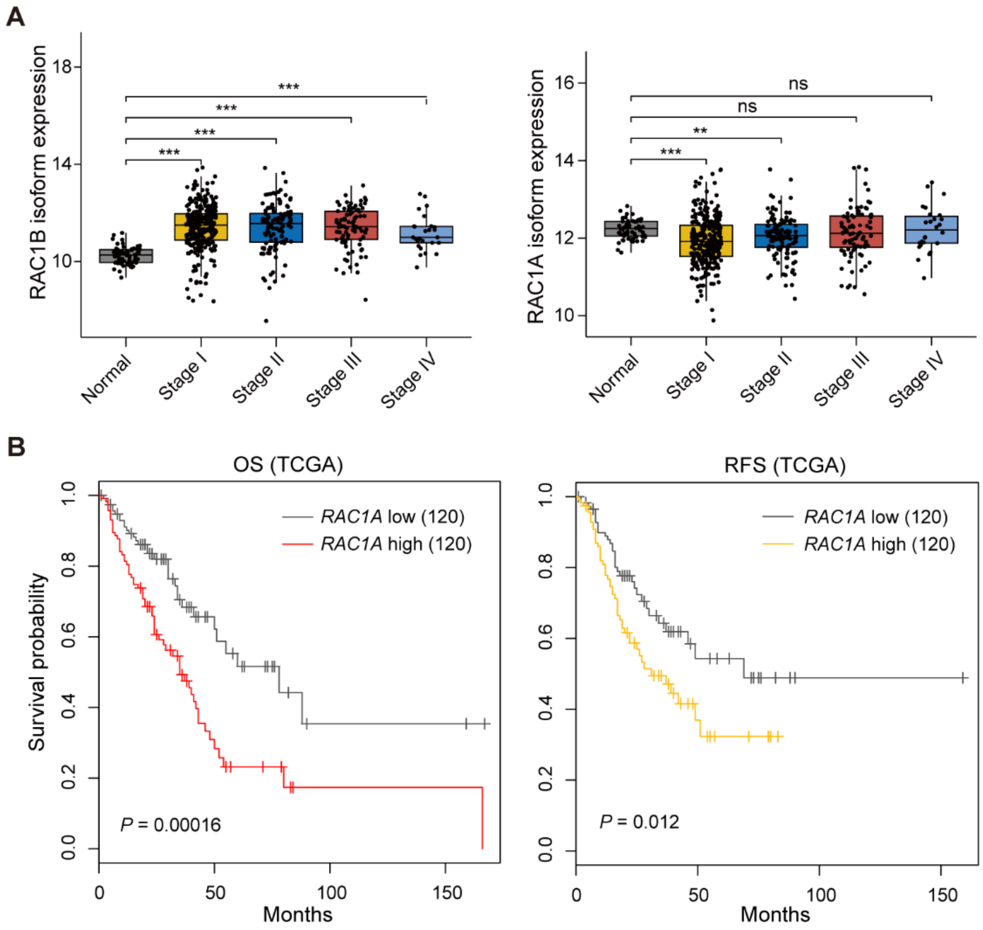

Supporting Figure S1. Expression and survival analyses for *RAC1A* and *RAC1B* isoform using TCGA LUAD datasets. (**A**) *RAC1A* and *RAC1B* isoform expression levels between non-tumor (NT) and tumor (T) tissues across different pathological stages of LUAD from the TCGA cohort. ** $P < 0.01$, *** $P < 0.001$, ns: not significant, one-way ANOVA with Dunnett's multiple comparison test. (**B**) Kaplan-Meier survival curves for overall survival (OS) and recurrence-free survival (RFS) in LUAD patients from the TCGA cohort stratified by high and low *RAC1A* expression levels. Log-rank test.

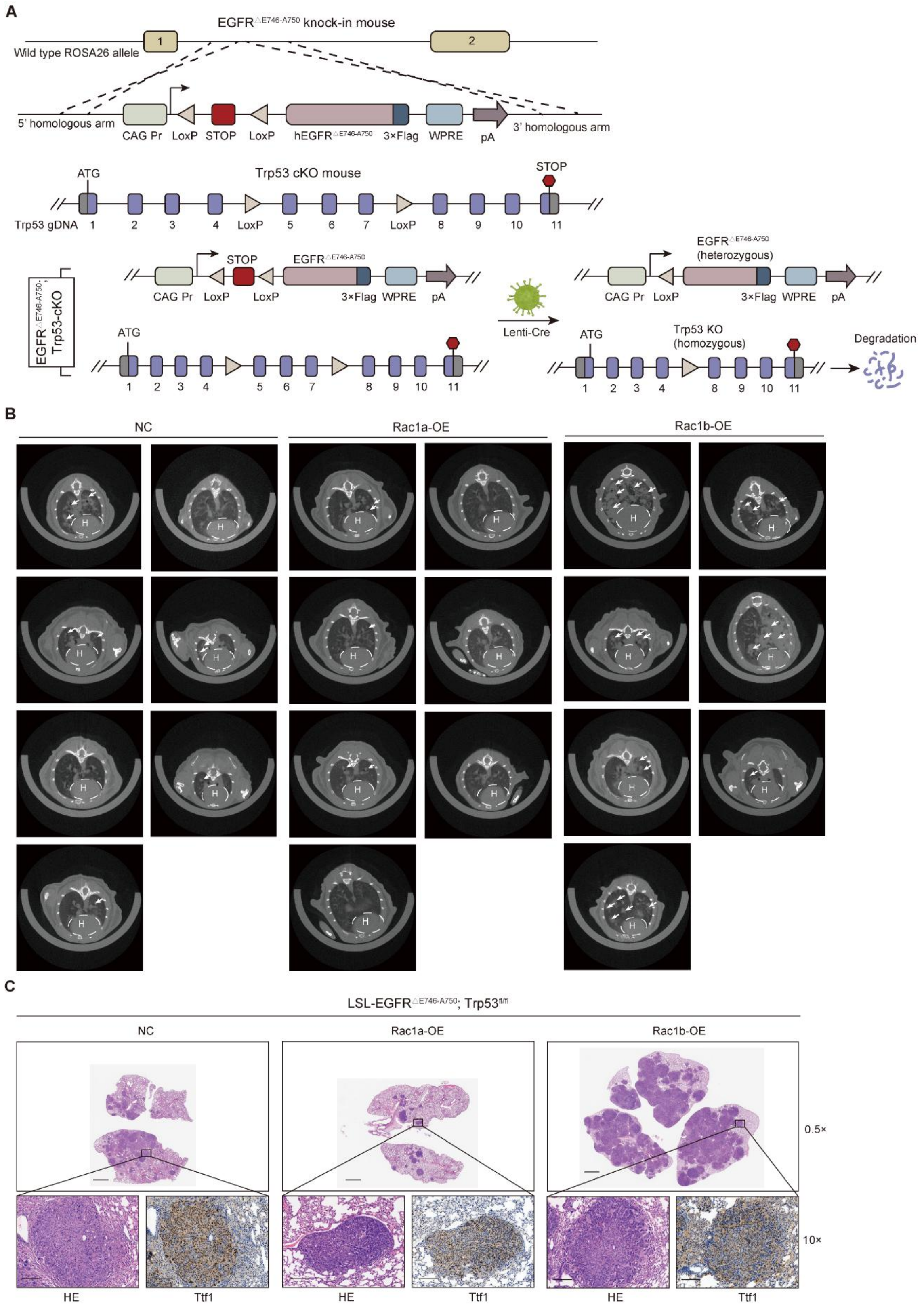
A
EGFR△E746-A750 knock-in mouse
Wild type ROSA26 allele
1
2
5' homologous arm
CAG Pr
LoxP
STOP
LoxP
hEGFR△E746-A750
3×Flag
WPRE
pA
3' homologous arm
Trp53 cKO mouse
ATG
STOP
Trp53 gDNA
1 2 3 4 LoxP 5 6 7 LoxP 8 9 10 11
EGFR△E746-A750; Trp53-cKO
CAG Pr LoxP STOP LoxP EGFR△E746-A750 3×Flag WPRE pA
Lenti-Cre
EGFR△E746-A750 (heterozygous)
CAG Pr LoxP 3×Flag WPRE pA
Trp53 KO (homozygous)
Degradation
B
NC
Rac1a-OE
Rac1b-OE
H
C
LSL-EGFR△E746-A750; Trp53fl/fl
NC
Rac1a-OE
Rac1b-OE
0.5×
10×
HE
Ttf1

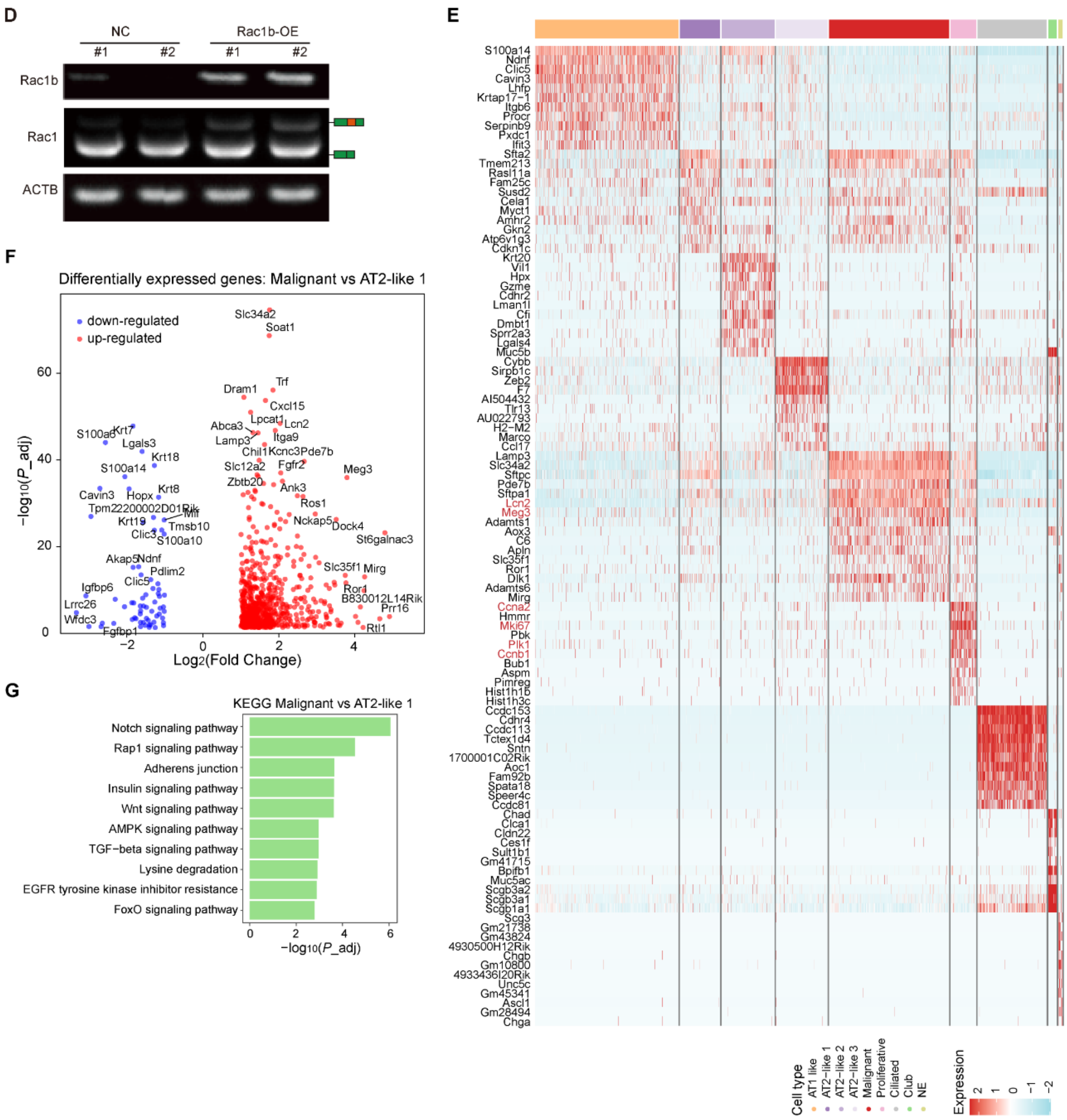

Supporting Figure S2. Lung specific overexpression of Rac1b but not Rac1a accelerates LUAD development in an *EGFR*-mutant murine model. (**A**) Schematic illustration of the generation of heterozygous EGFR$^{\Delta E746\text{-}A750}$ knock-in and homozygous *Trp53* knock-out mouse model. LSL: loxP-stop-loxP. (**B**) CT images of lung tumors from each mouse in NC (n=7), Rac1b overexpression (n=7) groups, and Rac1a overexpression (n=7) groups. (**C**) Representative images of H&E and Ttf-1 IHC staining of serial lung sections from NC, Rac1b-OE and Rac1a-OE mice. Scale bar = 50μm. (**D**) RT-PCR analysis of *RAC1B* exon 3b inclusion in representative NC and Rac1b OE lung tumors. Loading control: ACTB. (**E**) Heatmap of top differentially expressed genes (DEGs) across distinct clusters of epithelial cells. Rows represent individual genes, and columns represent cells

grouped by subtype. The color intensity reflects relative expression levels (log-normalized) from high (red) to low (blue). Specific marker genes in Malignant and Proliferative cells are highlighted. (**F**) Dot plot showing the differential gene expression analysis between Malignant and AT2-like 1 cells. Each point represents a gene, with red indicating significantly up-regulated genes and blue indicating significantly down-regulated genes in Malignant cells compared to AT2-like 1 cells. (**G**) KEGG pathway enrichment analysis of differentially expressed genes between Malignant and AT2-like 1 cells. The bar plot shows the selected top 10 significantly enriched KEGG pathways.

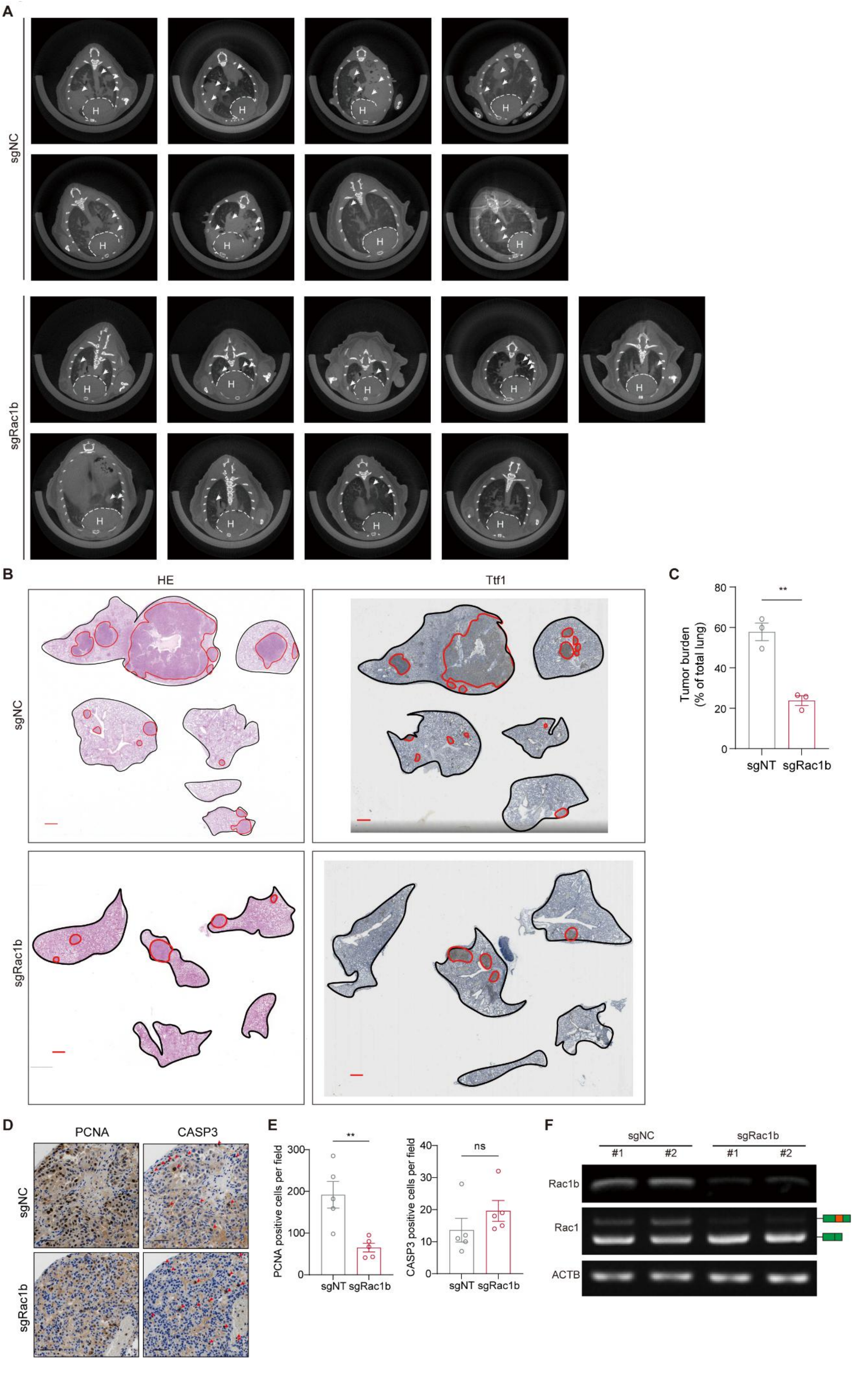

A
sgNC
H
sgRac1b
B
HE
Ttf1
sgNC
sgRac1b
C
Tumor burden
(% of total lung)
**
80
60
40
20
0
sgNT
sgRac1b
D
PCNA
CASP3
sgNC
sgRac1b
E
PCNA positive cells per field
**
300
200
100
0
sgNT
sgRac1b
CASP3 positive cells per field
ns
40
30
20
10
0
sgNT
sgRac1b
F
sgNC
sgRac1b
#1
#2
#1
#2
Rac1b
Rac1
ACTB

Supporting Figure S3. Lung specific depletion of *Rac1b* impedes LUAD development in an *EGFR*-mutant murine model. (**A**) CT images of lung tumors from each mouse in the sgNC (n=8) and sgRac1b (n=9) groups. (**B**) Representative images of hematoxylin and eosin (H&E) and TTF-1 immunohistochemistry (IHC) staining in sections of the whole lung from sgNC or sgRac1b mice. Scale bar = 1 mm. (**C**) Quantification of tumor burden based on the tumor foci indicated by H&E staining. n = 3 mice for each group. (**D**) Representative images of IHC staining of PCNA and cleaved caspase 3 (CASP3) in sections of tumors from sgNC or sgRac1b mice. Scale bar = 50 μm. (**E**) Quantification of positive cells in different fields as indicated in (D). (**F**) RT-PCR analysis of *RAC1B* exon 3b inclusion in representative sgNC or sgRac1b lung tumors. Loading control: ACTB.

** $P < 0.01$, *** $P < 0.001$, ns: not significant, Student's t-test in (C, E).

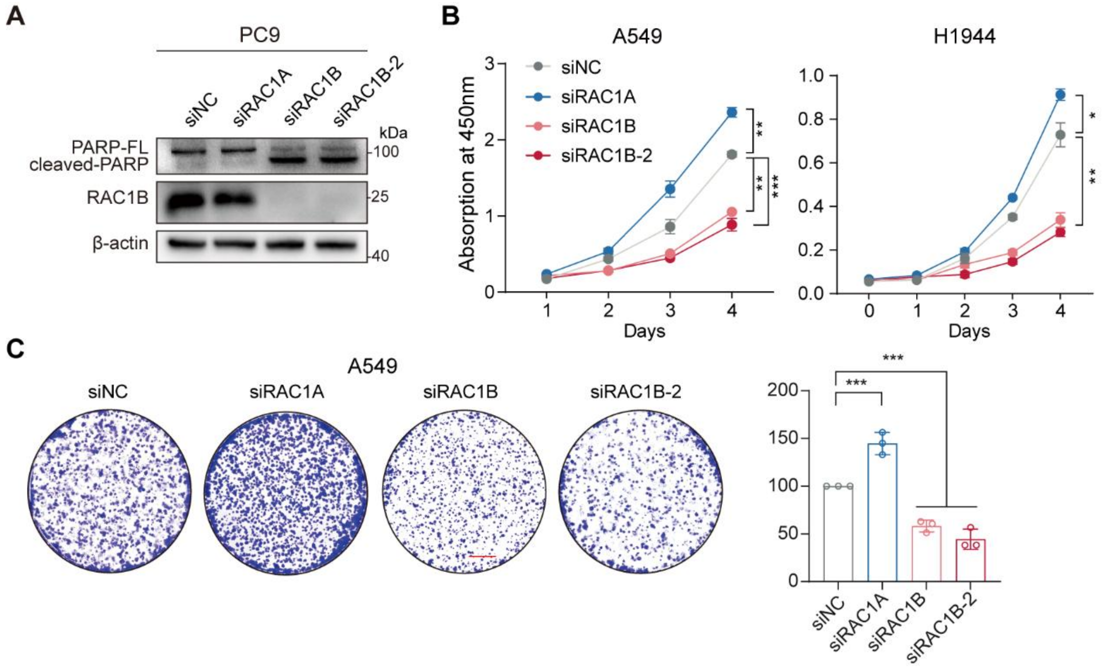

Supporting Figure S4. Effects of *RAC1A* or *RAC1B* knockdown in *EGFR*-mutant and *EGFR*–wild type cells. (**A**) Western blot detection of full length (FL) and cleaved-PARP following *RAC1A* or *RAC1B* knockdown in PC9 cells. (**B**, **C**) Effects of *RAC1A* or *RAC1B* knockdown on the proliferation of *EGFR*-wild type LUAD cells determined by CCK-8 (**B**) and colony formation (**C**) assays. n = 3 biological replicates, two-way ANOVA with Tukey's multiple comparison test in (B), and one-way ANOVA with Dunnett's multiple comparison test in (C). Scale bar: 1 cm.

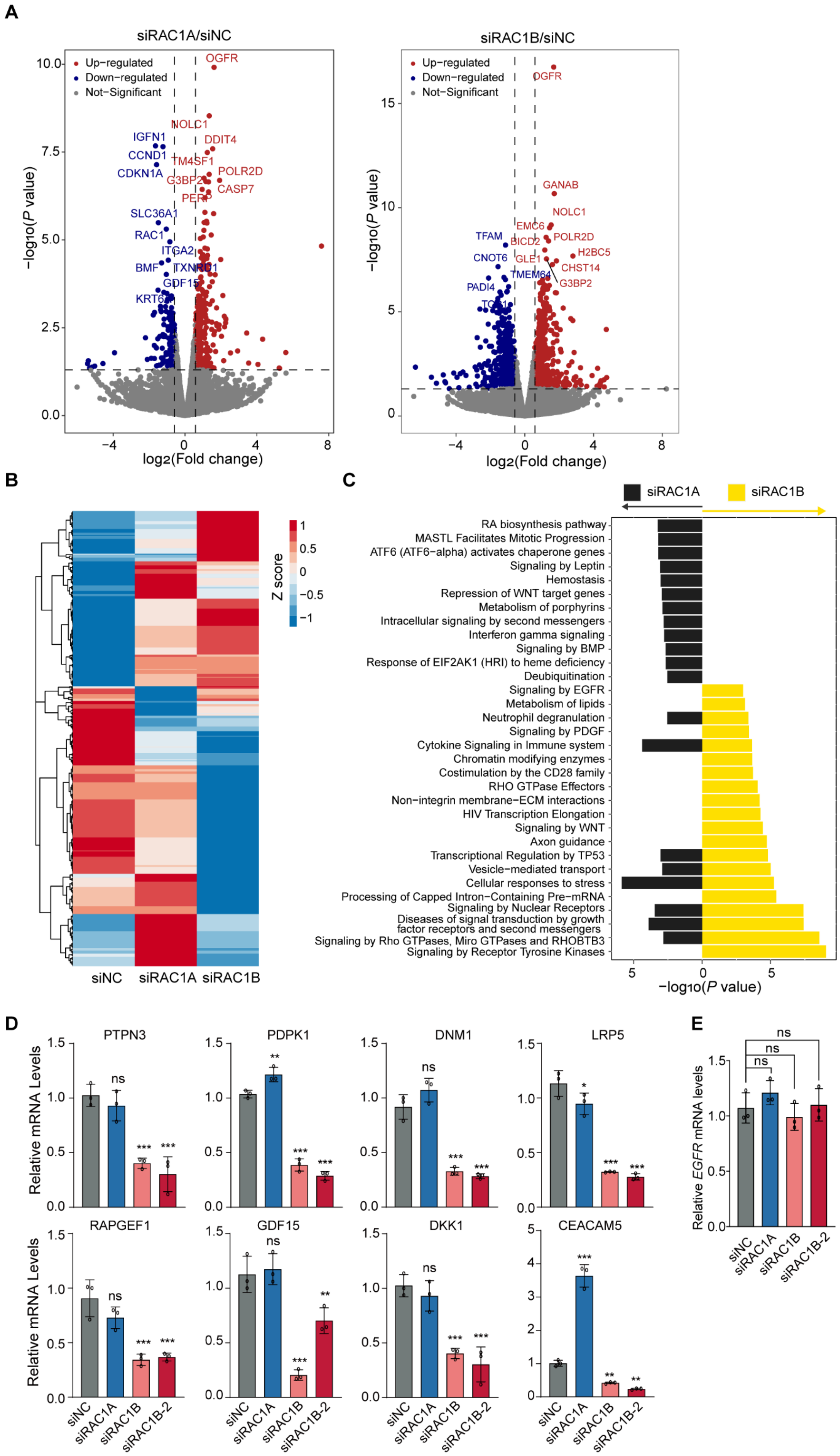
A
siRAC1A/siNC
Up−regulated
Down−regulated
Not−Significant
OGFR
NOLC1
DDIT4
IGFN1
CCND1
TM4SF1
CDKN1A
POLR2D
G3BP2
CASP7
PERP
SLC36A1
RAC1
ITGA2
BMF
TXNRD1
GDF15
KRT6A
−log10(P value)
log2(Fold change)
siRAC1B/siNC
GANAB
NOLC1
EMC6
BICD2
POLR2D
TFAM
H2BC5
CNOT6
GLE1
CHST14
TMEM64
G3BP2
PADI4
B
Z score
siNC
siRAC1A
siRAC1B
C
siRAC1A
siRAC1B
RA biosynthesis pathway
MASTL Facilitates Mitotic Progression
ATF6 (ATF6−alpha) activates chaperone genes
Signaling by Leptin
Hemostasis
Repression of WNT target genes
Metabolism of porphyrins
Intracellular signaling by second messengers
Interferon gamma signaling
Signaling by BMP
Response of EIF2AK1 (HRI) to heme deficiency
Deubiquitination
Signaling by EGFR
Metabolism of lipids
Neutrophil degranulation
Signaling by PDGF
Cytokine Signaling in Immune system
Chromatin modifying enzymes
Costimulation by the CD28 family
RHO GTPase Effectors
Non−integrin membrane−ECM interactions
HIV Transcription Elongation
Signaling by WNT
Axon guidance
Transcriptional Regulation by TP53
Vesicle−mediated transport
Cellular responses to stress
Processing of Capped Intron−Containing Pre−mRNA
Signaling by Nuclear Receptors
Diseases of signal transduction by growth factor receptors and second messengers
Signaling by Rho GTPases, Miro GTPases and RHOBTB3
Signaling by Receptor Tyrosine Kinases
−log10(P value)
D
PTPN3
PDPK1
DNM1
LRP5
RAPGEF1
GDF15
DKK1
CEACAM5
Relative mRNA Levels
siNC
siRAC1A
siRAC1B
siRAC1B-2
E
Relative EGFR mRNA levels
ns

Supporting Figure S5. Differential gene expression and pathway analysis of *RAC1A* and *RAC1B* knockdown in PC9 cell lines. (**A**) Volcano plots displaying DEGs after knockdown of *RAC1A* (left) and *RAC1B* (right) compared to negative control (siNC) in PC9 cell lines. siRAC1B group represents changes of DEGs shared by siRAC1B and siRAC1B-2. Genes significantly upregulated are shown in red, downregulated in blue, and non-significant genes in grey. (**B**) Heatmap showing the hierarchical clustering of DEGs after knockdown of RAC1A and RAC1B. Z-scores of expression levels are color-coded, with red indicating higher expression and blue indicating lower expression. (**C**) Bar plot representing the top enriched pathways in DEGs upon knockdown of *RAC1A* (black bars) and *RAC1B* (yellow bars) based on the Reactome database. (**D**) qPCR confirmation of expression changes of several cancer-related genes specific to *RAC1B* knockdown in PC9 cells. Error bars: ±SD. (**E**) qPCR analysis of *EGFR* mRNA expression following *RAC1A* or *RAC1B* knockdown in PC9 cells.

* $P < 0.05$, ** $P < 0.01$, *** $P < 0.001$, ns: not significant, one-way ANOVA with Dunnett's multiple comparison test in (D, E).

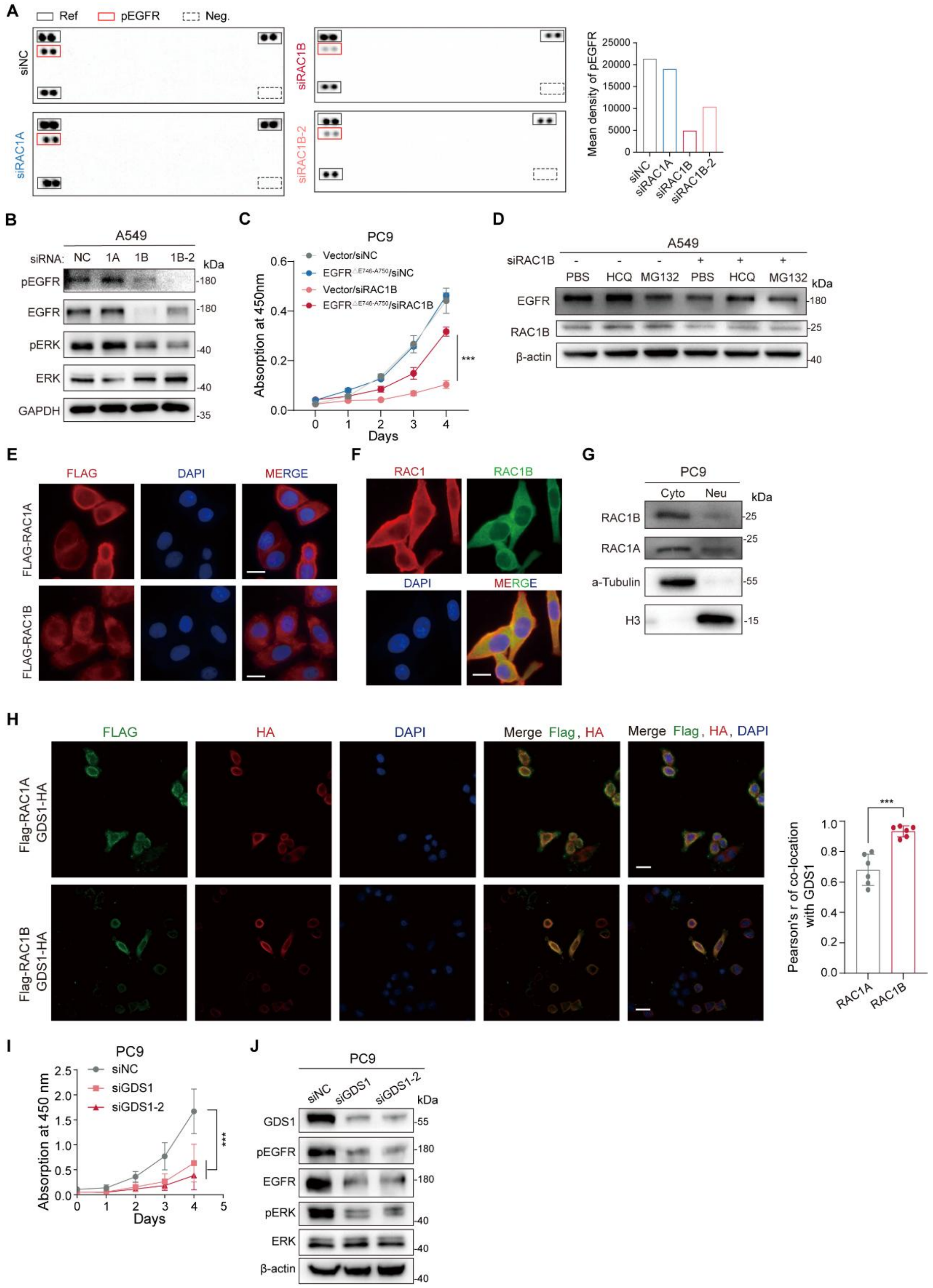

Supporting Figure S6. Effects of RAC1B and its interactor GDS1 on EGFR protein expression. (**A**) Phospho-receptor tyrosine kinase arrays for *RAC1A* knockdown (siRAC1A), *RAC1B* knockdown (siRAC1B, siRACB-2) in osimertinib-resistant H1975 (H1975-OR) cells. Phosphorylated EGFR (pEGFR) is highlighted with red boxes.

Quantification of pEGFR level is presented at the right part of the panel. (**B**) Western blot analysis of the phosphorylated and total EGFR and ERK in A549 cells following *RAC1A* or *RAC1B* knockdown. Loading control: β-actin. (**C**) Cell proliferation of control and *RAC1B* knockdown in PC9 cells without or with the EGFR mutant overexpression. n = 3 replicates. (**D**) Western blot analysis of EGFR protein expression in control and *RAC1B*-silenced A549 cells treated with DMSO control, HCQ (50μM, 24h) or MG132 (10μM, 24h). Quantification of EGFR expression was shown at the part of the panel. Loading control: β-actin. (**E**) Immunofluorescence assay and confocal images of PC9 cells stably expressing FLAG-RAC1A or FLAG-RAC1B. Scale bar: 15 μm. (**F**) Immunofluorescence assay of endogenous RAC1 and RAC1B in PC9 cells. Scale bar: 15 μm. (**G**) Western blot analysis of indicated proteins following nucleus and cytosol fractionation in PC9 cells. (**H**) Confocal images of PC9 cells stably co-expressing FLAG-RAC1A (green) or FLAG-RAC1B (green) and HA-GDS1 (red). Quantification of colocalized HA and FLAG by the Pearson correlation coefficient is shown at the right part of the panel. Scale bar: 25μm. n = 3 biological replicates, *** $P < 0.001$. (**I, J**) Cell proliferation measured by CCK-8 (**I**) and Western blot analysis of indicated proteins (**J**) in PC9 cells following *GDS1* silencing. n = 3 biological replicates.

Error bar: ±SD, *** $P < 0.001$, two-way ANOVA with Tukey's multiple comparison test in (C, H), Student's t-test in (G).

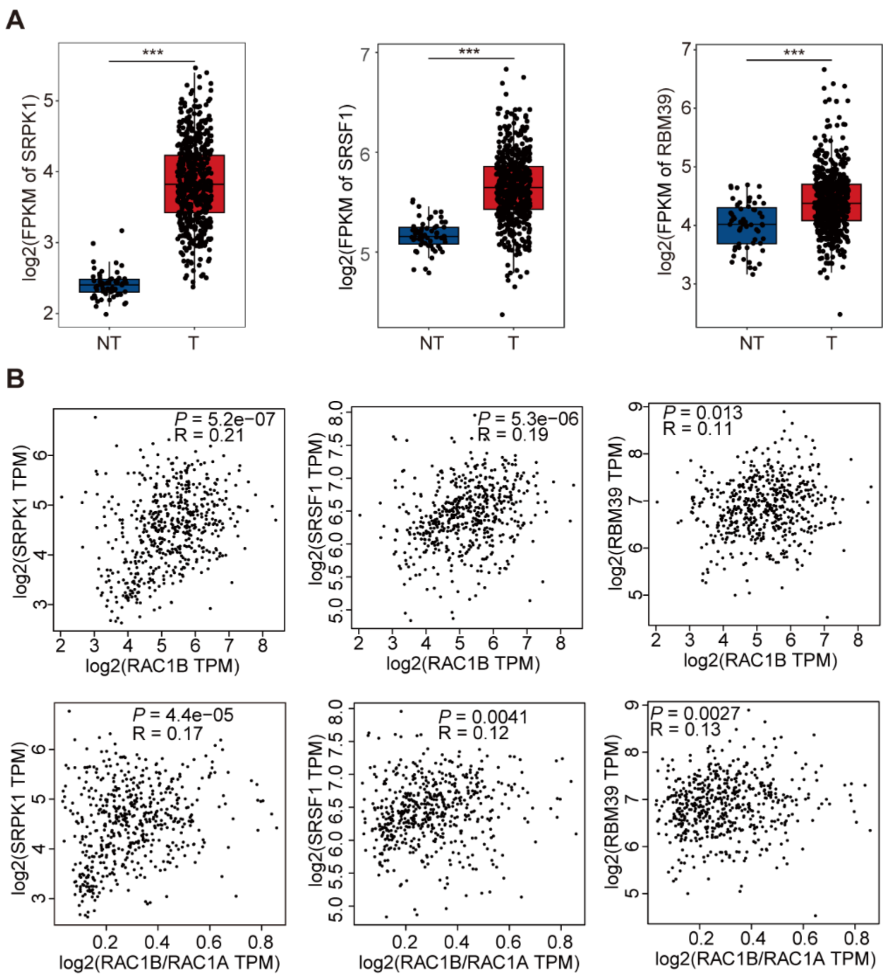

Supporting Figure S7. Expression changes of the three positive splicing regulators of RAC1B and their correlation with *RAC1B* expression in TCGA LUAD. (**A**) Relative expression of *SRPK1*, *SRSF1* and *RBM39* in adjacent non-tumor and tumor tissues of LUAD samples from TCGA. FPKM: fragments per kilobase of transcript per million fragments mapped. *** $P < 0.001$, Wilcoxon test. (**B**) Correlation between expression of *SRPK1*, *SRSF1* or *RBM39* and *RAC1B* (upper panel) or *RAC1B/RAC1A* (lower panel) based on TCGA LUAD tumor and adjacent non-tumor data. Plots were generated by GEPIA 2 (http://gepia2.cancer-pku.cn/#index).

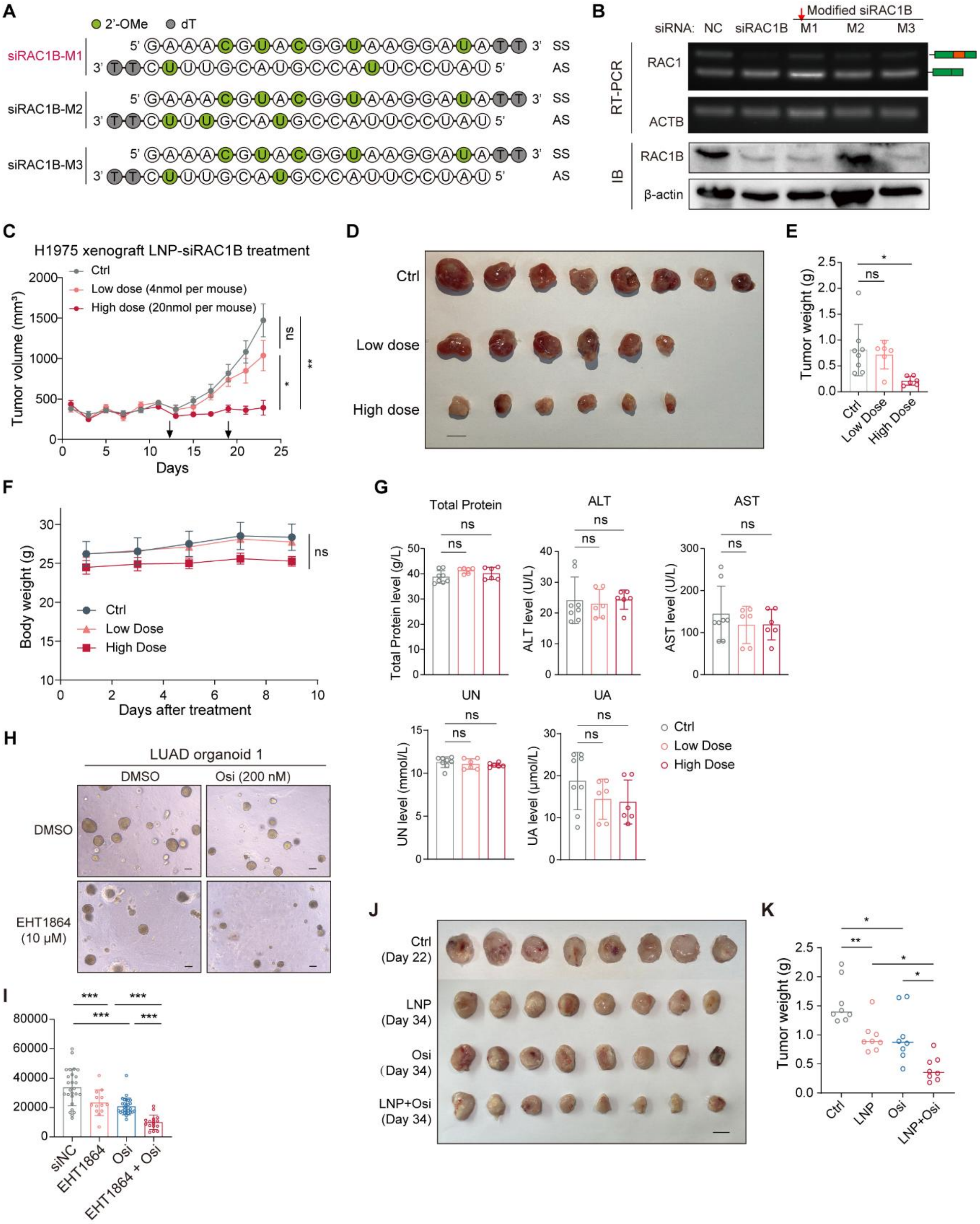

Supporting Figure S8. Pharmacological inhibition of RAC1B suppresses LUAD tumor growth. (**A**) Experimental scheme of 2-methoxyethyl (2-OME) modification of cytosine and uracil in siRAC1B. Briefly, cytosine and uracil on the sense strand are modified with 2-OME, while those on the antisense strand are selectively modified. The bases modified with 2-OME are highlighted with green circles. (**B**) Knockdown efficiency of unmodified siRAC1B or modified siRAC1B (M1，M2 or M3) was examined by RT-PCR

and Western blot analysis. Loading control: ACTB for RT-PCR and β-actin for Western blot. (**C-G**) H1975 cells were transplanted in lower flanks of BALB/c-nude mice and subsequently treated with negative control, low dose lipid nanoparticle packaged siRAC1B (LNP-siRAC1B, 4nmol per mouse) or high dose LNP-siRAC1B (20nmol per mouse). Growth curve (**C**), end point illusion (**D**), tumor weight (**E**), mouse body weight (**F**) as well as markers of liver and kidney function in NC (n = 8), low dose (n = 6), and high dose (n = 6) groups. Error bar: ±SEM in (C). ALT: alanine aminotransferase, AST: aspartate aminotransferase, UN: urea nitrogen, UA: uric acid. (**H, I**) Morphology (**H**) and quantification of relative areas (**I**) of PDOs treated with vehicle control or EHT1864 and Osi alone or in combination. Scale bar =50μm. (**J, K**) End point illusion (**J**) and tumor weight (**K**) of PDX01 with treatments as indicated in Fig. 6L. Scale bar: 1 cm.

Error bars represent ±SD unless indicated, * $P < 0.05$, ** $P < 0.01$, *** $P < 0.001$, ns: not significant, one-way ANOVA with Dunnett's multiple comparison test in (E, G, I, K), and two-way ANOVA with Tukey's multiple comparison test in (C, F).

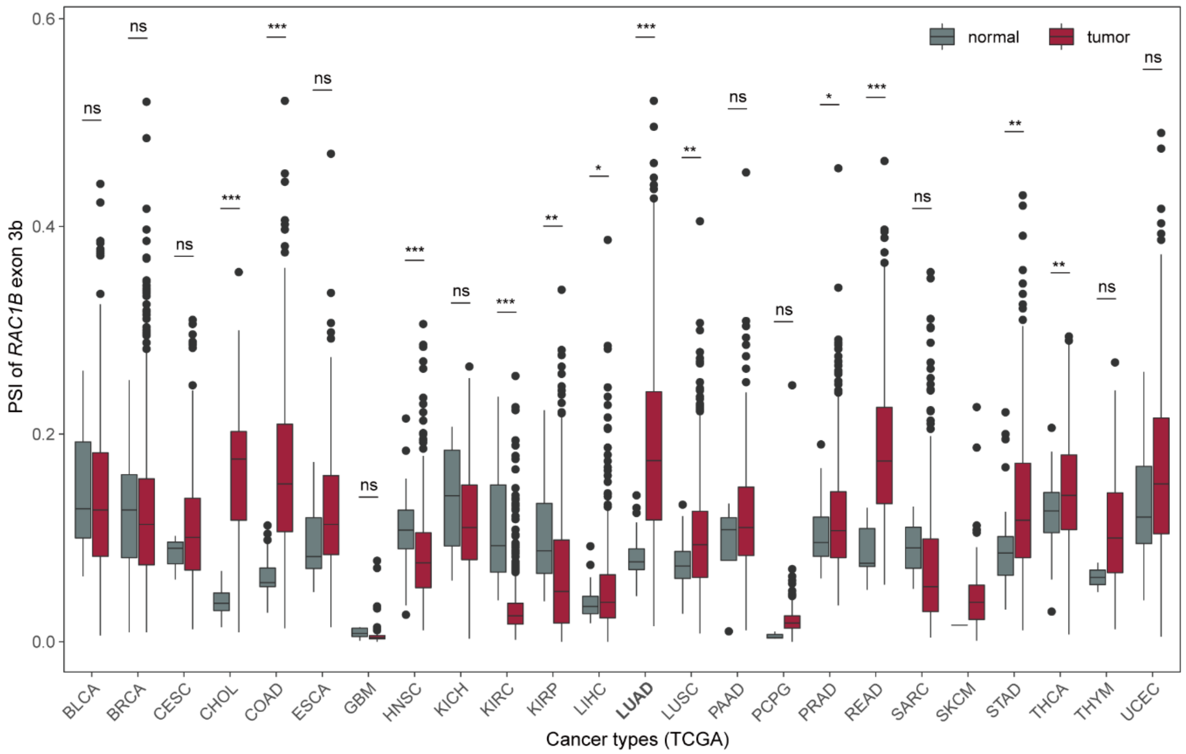

Supporting Figure S9. *RAC1B* exon 3b inclusion levels in various cancer types.

Data from TCGA. * *P* < 0.05, ** *P* < 0.01, *** *P* < 0.001, ns: not significant, two-way ANOVA with Tukey's multiple comparison test in (C), and Wilcoxon test in (D).
